# Photoluminescence Properties of Two Closely Related Isostructural Series Based on Anderson-Evans Cluster Coordinated With Lanthanides [Ln(H_2_O)_7_{X(OH)_6_Mo_6_O_18_}]•yH_2_O, X = Al, Cr

**DOI:** 10.3389/fchem.2018.00631

**Published:** 2019-01-07

**Authors:** Shailabh Tewari, Mohammad Adnan, Balendra, Vineet Kumar, Gaurav Jangra, Gaddam Vijaya Prakash, Arunachalam Ramanan

**Affiliations:** ^1^Department of Chemistry, Indian Institute of Technology Delhi, New Delhi, India; ^2^Department of Physics, Indian Institute of Technology Delhi, New Delhi, India

**Keywords:** Anderson-Evans cluster, structural chemistry, lanthanides, chromium molybdate, aluminum molybdate, photoluminescence

## Abstract

The paper describes synthesis and structural characterization of the whole series of two closely related lanthanide coordinated chromium or aluminum hexamolybdates (Anderson-Evans cluster) including twelve new members hitherto unreported: [Ln(H_2_O)_7_{X(OH)_6_Mo_6_O_18_}]·4H_2_O and [Ln(H_2_O)_7_{X(OH)_6_Mo_6_O_18_}Ln(H_2_O)_7_]{X(OH)_6_Mo_6_O_18_}·16H_2_O where X = Al or Cr and Ln = La, Ce, Pr, Nd, Sm, Eu, Gd, Tb, Dy, Ho, Er, Tm, Yb, Lu, and Y. Crystal structures of all the solids were established by powder and single crystal X-ray diffraction techniques. The two series are dictated by a different aggregation of the same set of molecular species: Lighter lanthanides favor coordination interaction between lanthanide ions and molybdate cluster forming 1D chains (Series I) while the heavier lanthanides result in the stacking of a cation, a pair of lanthanide hydrates coordinating to the cluster, and an anion, the discrete cluster is further stabilized through a large number of water molecules (Series II). Crystallization with Er^3+^ and Tm^3+^ ions results in a concomitant mixture of Series I and II. Photoluminescence of single crystals of all the chromium molybdates was dominated by a ruby-like emission including those which contain optically active ions Pr, Sm, Eu, Tb, Dy, and Tm. In contrast, aluminum analogs showed photoluminescence corresponding to characteristic lanthanide emissions. Our results strongly suggest a possible energy transfer from *f* levels of lanthanide ions to *d* levels of chromium (III) causing the quenching of lanthanide emission when coordinated with chromium molybdates. Intensity measurements showed that the emission from chromium molybdates are almost two orders of magnitude lower than naturally occurring ruby with broader line widths at room temperature.

## Introduction

**Figure 1:**
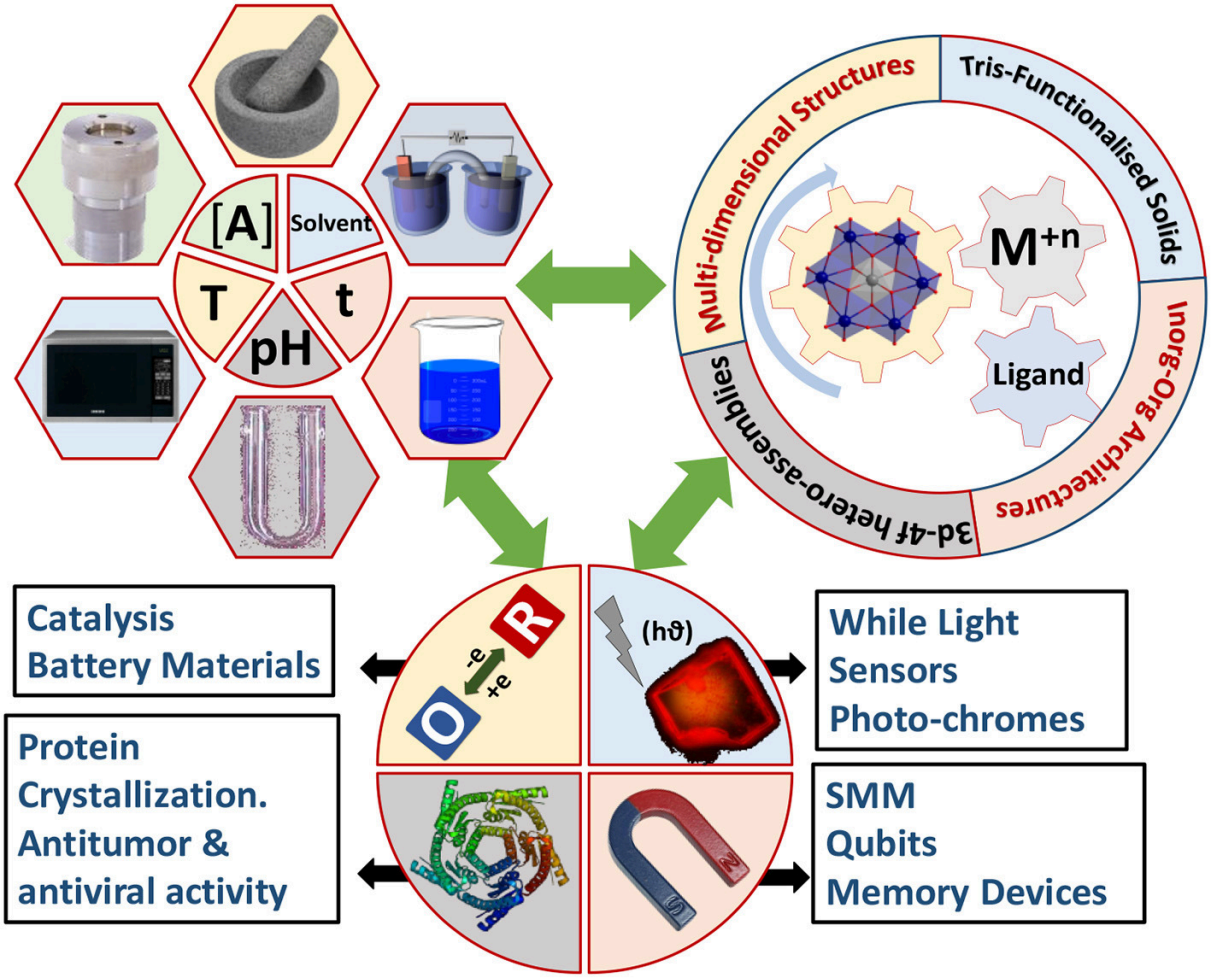
The Synthesis-Structure-Property correlations leading to multiple applications in M^n+^-Anderson-Evans system.

**Figure 2:**
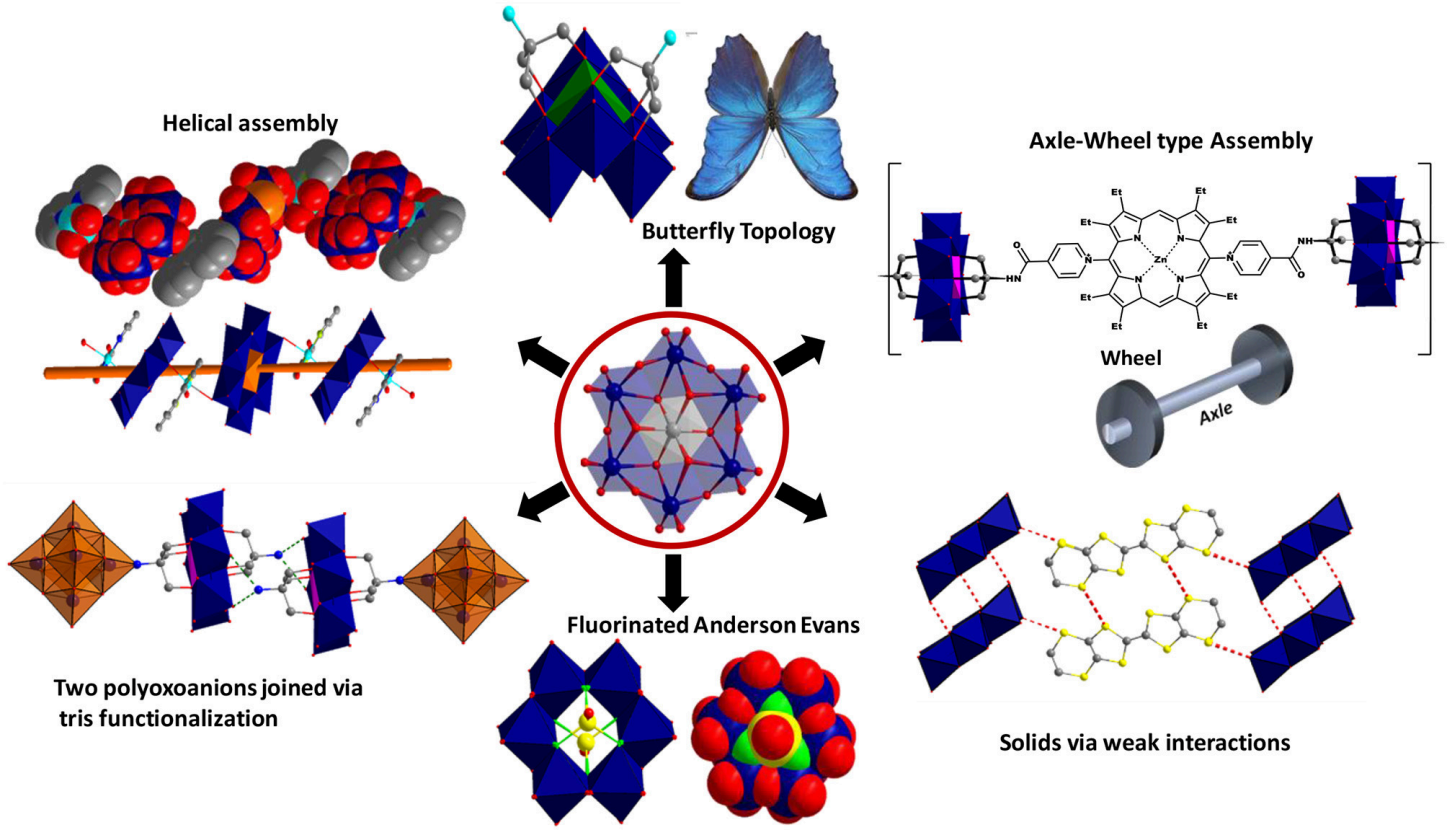
The structural versatility in Anderson-Evans cluster based solids.

Polyoxomolybdates (POM), an important branch in polyoxometalate chemistry, presents an unrivaled structural chemistry and physicochemical properties providing immense opportunities as well as considerable challenges in creating new functional materials (Hill, 1998; Pope, 2002; Li and Xu, 2011; Eldik and Cronin, 2017; Song, 2018). Among the POM anions, a hetero polyoxomolybdate of the composition, [X^n+^Mo_6_O_24_H_y_]^(12−n−y)−^, was found to be a dominant building block in several solids (Blazevic and Rompel, 2016). Its molecular structure, first proposed almost eighty years ago (Anderson, 1937), was established experimentally by refining the X–ray crystallographic data obtained for the tellurium salts, (NH_4_)_6_{TeMo_6_O_24_}·7H_2_O and K_6_{TeMo_6_O_24_}·7H_2_O (Evans, 1948). Anderson-Evans cluster, as it has been known since, is made of six edge-shared distorted {MoO_6_} octahedra and the central cavity thus formed is occupied by slightly flattened, X(OH)_6_, heteroatom octahedron. The cluster assumes a near D_3d_ symmetry with dimensions ~8.6 × 8.6 × 2.7 Å and has been isolated with fifteen different heteroatoms till now [X: **Al** (Lee et al., 1991; Manikumari et al., 2002; Shivaiah et al., 2002; Martin et al., 2004; Shivaiah and Das, 2005; Liu et al., 2006; Dhara et al., 2007; Cao et al., 2008; Zhou et al., 2009), **Cr** (Perloff, 1970; An et al., 2006; Yu et al., 2006; Lee, 2007; Zhou and Yang, 2007; Shi D. et al., 2008; Shi D. M. et al., 2008; Zhang et al., 2008, 2014; Singh et al., 2010b, 2014; Li et al., 2011; Singh and Ramanan, 2011; Kumar et al., 2014a; Joo et al., 2015), **Mn** (He et al., 2012; Oms et al., 2012; Rosnes et al., 2012, 2013; Yan et al., 2012; Zhang et al., 2012, 2015a,b; Hutin et al., 2013; Ai et al., 2014; Yvon et al., 2014), **Fe** (Wu et al., 2001), **Co** (Nolan et al., 1996; Panneerselvam et al., 1996; Lee and Joo, 2000a; Lee et al., 2001; Martin et al., 2004), **Ni** (Lee et al., 2002; Liu et al., 2007; Liu F.-X. et al., 2008), **Cu** (Ito et al., 1989), **Zn** (Allen et al., 1997), **Ga** (Mensinger et al., 2008; Himeno et al., 2009), **Te** (Evans, 1948; Kondo et al., 1980; Robl and Frost, 1993a,b; Lorenzo-Luis et al., 1999; Drewes et al., 2004a,b; Charushnikova et al., 2005; Drewes and Krebs, 2005; Gao et al., 2007; Liu Y. et al., 2008; Yan et al., 2008), **Rh** (Ozawa et al., 1991), **Pd** (Ozawa et al., 1991), **Sb** (Ogawa et al., 1988; Yan et al., 2014), **I** (Kondo et al., 1980; Rosu and Weakley, 2000; An et al., 2005)and **Pt** (Lee and Sasaki, 1984; Lee, 1994; Lee and Joo, 2000b, 2004, 2006a,b, 2007)]. The “disc-shaped” geometry and the nucleophilicity of the twelve terminal oxygens (O_t_) render the POM cluster as an attractive ligand to engineer helices (Shivaiah et al., 2003), stacks or multidimensional frameworks (An et al., 2005; Ritchie and Bryant, 2015) through hydrogen bonding as well as metal-oxygen coordination. Furthermore, the structural versatility of this cluster is unique and can be tuned at the molecular level. It can be modified through the substitution of the triply bridged OH groups with fluoride ions (Michailovski et al., 2009), “axle-wheel” type (Schaming et al., 2010; Allain et al., 2013) with appropriate ligands or bent “butterfly” topologies (Zhang et al., 2017) via suitable functionalization (Figure 2). For this reason, a range of applications, from the much studied luminescent (Ohashi et al., 1982; Yamase and Sugeta, 1993; Ito et al., 2006; Kumar et al., 2014a), catalytic (Tanaka et al., 2012; Bayraq et al., 2013), anti-tumor (Yamase, 1993; Raza et al., 2012; Shah et al., 2014), anti-viral (Inouye et al., 1993), photochromic (Coué et al., 2007; Song et al., 2008; Pardo et al., 2011; Oms et al., 2012; Allain et al., 2013; Hakouk et al., 2014) and magnetic (Leuenberger and Loss, 2001; Lehmann et al., 2007; Kushch et al., 2009; Wu et al., 2009; Feng et al., 2012) to the recently realized macromolecular crystallography (Bijelic et al., 2014; Mauracher et al., 2014a,b; Bijelic and Rompel, 2015) and functional bio-nanomaterials (Massia and Hubbell, 1991; Aota et al., 1994; Song et al., 2009) are envisaged for solids based on it (Figure 1). Synthesis-structure-function correlation is key to recognize the utility of these solids as futuristic materials.

There has been a renewed interest in the synthesis of lanthanide-based coordination solids with multidimensional structures; the trend is to tune the size, shape and dimensionality between nano to micro level to optimize luminescent properties for a desirable purpose (Binnemans, 2009; Armelao et al., 2010; Bünzli and Eliseeva, 2013; de Bettencourt-Dias, 2014). In this context, a few groups have investigated photoluminescence (PL) of lanthanide-Anderson-Evans cluster based solids (Yusov et al., 2002; Cao et al., 2008; Shi D. M. et al., 2008; Yang et al., 2013; Kumar et al., 2014a). The solid [Eu(H_2_O)_7_{Cr(OH)_6_Mo_6_O_18_}]·4H_2_O belonging to Series I showed only characteristic emission due to Eu^3+^ ions (Shi D. M. et al., 2008). The solid [(C_6_H_5_NO_2_)_2_Dy(H_2_O)_6_{Cr(OH)_6_Mo_6_O_18_}]·2C_6_H_5_NO_2_·6H_2_O showed characteristic emission due to Dy ^3+^ ion (Yang et al., 2013)). Dinesh et al. observed that Sm^3+^ and Tb^3+^ members of Series I showed only a red emission (Kumar et al., 2014a) reminiscent of ruby, while the lanthanide ion remained optically silent. Another molecular solid containing no lanthanide ions, [(Hbipy)_2_{Cr(OH)_6_Mo_6_O_17_(OH)}(bipy)] also showed a ruby-like emission (Li et al., 2016). It should be mentioned that Yamase and his group were the first to observe the red emission reminiscent of ruby (Yamase and Sugeta, 1993; Ito et al., 2006) in Na_3_[Cr(OH)_6_Mo_6_O_18_]·8H_2_O at 4.2 K. It was noted that majority of the chromium hexamolybdate based crystals reported in literature, including the aforementioned solids, appeared pink in color. This suggested the domination of red emission arising from chromium ion. The contradicting photoluminescent behavior raised an obvious question, whether the presence or absence of a coordination linkage between the chromium molybdate cluster and lanthanide ions has any influence on their optical properties. This prompted us to explore the structural landscape of the system Ln^3+^_(aq)_-{X(OH)_6_Mo_6_O_18_}-H_2_O (X = Cr^3+^ or Al^3+^).

A structural analysis of the reported solids with the general composition [Ln(H_2_O)_7_{X(OH)_6_Mo_6_O_18_}]·yH_2_O, in particular, showed the occurrence of two types: (i) Formation of 1D chains between hydrated {Ln(H_2_O)_n_} and the cluster through extended –Ln–(O_t_-cluster-O_t_)–Ln– leading to **Series I** (Figure S1) of the composition [Ln(H_2_O)_7_{X(OH)_6_Mo_6_O_18_}]·4H_2_O and (ii) occurrence of a molecular solid (0D) resulting from an aggregation between two discrete units, cationic [Ln(H_2_O)_7_{X(OH)_6_Mo_6_O_18_}Ln(H_2_O)_7_]^3+^ and anionic {X(OH)_6_Mo_6_ O_18_}^3−^ crystallizing into **Series II** (Figure S2) of the formula [Ln(H_2_O)_7_Ln{X(OH)_6_Mo_6_O_18_}Ln(H_2_O)_7_] {X(OH)_6_Mo_6_O_18_}·16H_2_O. The lack of systematic synthetic protocol in literature underlined the need to adopt a uniform procedure to crystallize the complete series of solids [Ln(H_2_O)_7_{Cr(OH)_6_Mo_6_O_18_}]·xH_2_O with an objective to understand the crystal structures, the origin of its photophysical properties and establish a structure-property correlation. For comparison, we also synthesized the same series with optically inactive aluminum as heteroatom, though a few members have already been reported. Our study has established the growth of the two structurally related members, Series I and II, with similar composition (except for the number of lattice water) as we go along the period. The decrease in size of the lanthanides favors a coordination change from nine to eight and is thus responsible for triggering a slightly different supramolecular assembly. The paper reports the synthesis, structure and photoluminescence properties of the whole series including twelve new members, to the best of our knowledge, hitherto unreported: [Ln(H_2_O)_7_{X(OH)_6_Mo_6_O_18_}]·4H_2_O, X = Al and Ln = Pr, Nd, Tb, Dy and Ho; X = Cr and Ln = Tb, Dy and Ho; [Ln(H_2_O)_7_{X (OH)_6_Mo_6_O_18_}Ln(H_2_O)_7_]{X(O H)_6_Mo_6_O_18_}·16H_2_O, X = Al and Ln = Er, Tm and Lu, X = Cr and Ln = Lu.

## Experimental

Selectedmembersofthecomposition [Ln(H_2_O)_7_{X(OH)_6_Mo_6_O_18_}]·4H_2_O and[Ln(H_2_O)_7_{X(OH)_6_Mo_6_O_18_}Ln(H_2_O)_7_]{X(OH)_6_Mo_6_O_18_}·16H_2_O have been isolated earlier by a few groups and its single crystal structures have been established (Fedoseev et al., 2002; Shivaiah et al., 2002, 2014; Gavrilova and Molchanov, 2005; Zhou and Yang, 2007; Cao et al., 2008; Shi D. et al., 2008; Shi D. M. et al., 2008; Zhang et al., 2008; Zhao et al., 2008; Wang et al., 2011; Kumar et al., 2014a). However, no group has attempted isolation of the whole series of Anderson-Evans cluster based solids containing Al or Cr heteroatoms coordinated to lanthanide cations. Two different approaches have been employed in literature to prepare the compounds: (a) Mixing aqueous solutions of pre-synthesized Na_3_Cr(OH)_6_Mo_6_O_18_·8H_2_O with aqueous (or methanolic) solutions of lanthanide salts followed by heating or refluxing to 60–80°C for 30 min to 4 h after suitably adjusting *p*H of the reacting mixture (Zhou and Yang, 2007; Shi D. et al., 2008; Shi D. M. et al., 2008; Zhao et al., 2008; Wang et al., 2011); (b) Reacting aqueous solutions of precursor compounds for the cluster (Na_2_MoO_4_·2H_2_O and CrCl_3_·6H_2_O or Cr(NO_3_)_3_·9H_2_O/AlCl_3_·6H_2_O) with an acidified solution of lanthanide salts. *p*H of the reaction mixture was then set before keeping for solvent evaporation. In some cases the reaction mixture was refluxed at 60°C prior to solvent evaporation (Shivaiah et al., 2002, 2014; Cao et al., 2008; Kumar et al., 2014a). Our earlier experience with the synthesis of the chromium molybdate based solids (Singh et al., 2010b, 2014; Pavani et al., 2011; Singh and Ramanan, 2011; Kumar et al., 2014a) showed the quality of crystals and phase homogeneity varied considerably depending on the concentration and *p*H of the reacting mixtures and the sequence of the addition of reagents. Although stoichiometry is generally the key to synthesis, in selected cases, excess or control of heteroatom concentration was found to be necessary (Pope, 1983). We also noticed that the procedures employed by other groups were limited leading to low yields and problems associated with the pre-synthesized sodium salt of Anderson-Evans cluster that resulted in competing phases driven by sodium hydrates. In aqueous solution, sodium ions are known to assemble differently with {Cr(OH)_6_Mo_6_O_18_}^3−^ leading to multiple forms (Perloff, 1970; Yu et al., 2006; Singh et al., 2010b).

This led us to adopt a modified method to prepare new {Cr(OH)_6_Mo_6_O_18_} based solids coordinated with transition metal ions (Singh et al., 2010b). An extension of this one-pot scheme did yield some success in isolating a few lanthanide-based chromium hexamolybdates (Kumar et al., 2014a). The method, however, was hampered to extend the series with other lanthanide ions due to the appearance of a green colored residue, possibly the formation of the solid [{Na(H2O)_6_}{Cr(OH)_6_Mo_6_O_17_(OH)}]·24H_2_O (Singh et al., 2010b; Pavani et al., 2011). Furthermore, these crystals started growing when the supernatant solution was left for longer time. Our initial attempts to prepare aluminum analogs led to two types of crystalline solids [Ln(H_2_O)_7_{Al(OH)_6_Mo_6_O_18_}·4H_2_O] (smaller block shaped transparent crystals) and [Na_3_(H_2_O)_6_{Al(OH)_6_Mo_6_O_18_}·2H_2_O] (larger block shaped transparent-white crystals). To overcome these issues, we lowered the concentration of CrCl_3_·6H_2_O and AlCl_3_·6H_2_O and the amount of acetic acid used to adjust *p*H as per our previous experience. The following modified protocol has enabled us to successfully isolate almost all lanthanide-based chromium and aluminum analogs of Anderson-Evans cluster solids.

**Modified synthetic protocol:**

The solid AlCl_3_·6H_2_O and all lanthanide salts (99.9% purity, trace metal basis) were purchased from Sigma Aldrich Chemicals Pvt. Ltd. (USA). Na_2_MoO_4_·2H_2_O was purchased from TCI Pvt. Ltd. and CrCl_3_·6H_2_O was purchased from CDH Pvt. Ltd. All reagents were used without further purification. Initially, two separate solutions were prepared as per our earlier method (Singh and Ramanan, 2011).

**Solution A** was prepared by dissolving 0.65 mmol of Na_2_MoO_4_·2H_2_O and 0.26 mmol CrCl_3_·6H_2_O/AlCl_3_·6H_2_O in 4 ml distilled water which was further acidified with 1 ml glacial CH_3_COOH. **Solution B** was prepared by dissolving 0.65 mmol LnCl_3_·xH_2_O (*x* = 7 for La and Ce; *x* = 6 for Nd to Er, Yb, Lu and Y) or Ln(NO_3_)_3_·xH_2_O (*x* = 6 for Pr and *x* = 5 for Tm) in 5 ml distilled water. The contents of solution A and B were mixed with continuous stirring and the resultant solution was kept at room temperature for solvent evaporation. In the case of lanthanum and cerium, crystals grew within 24 h. However, heavier lanthanides took more time to crystallize. Ytterbium crystals appeared after about 10 days.

### Crystal Structures

**Figure 3:**
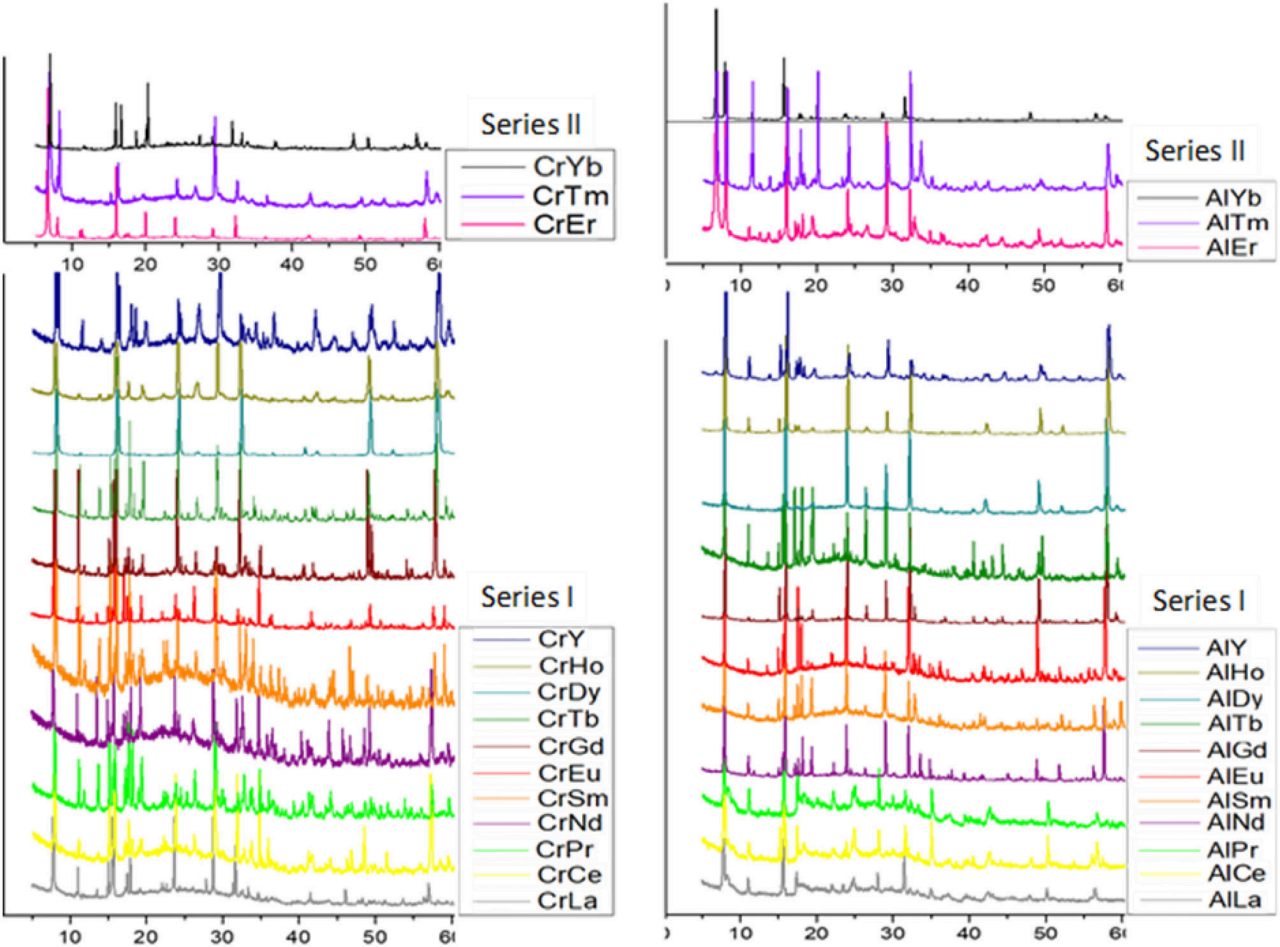
Powder X-ray diffraction patterns of series I, [Ln(H_2_O)_7_{X(OH)_6_Mo_6_O_18_}]·4H_2_O, Ln = Y, La, Ce, Pr, Nd, Sm, Eu, Gd, Tb, Dy, and Ho and series II, [Ln(H_2_O)_7_{X(OH)_6_Mo_6_O_18_}Ln(H_2_O)_7_]{X(OH)_6_Mo_6_O_18_}·16H_2_O, Ln = Er, Tm and Yb. {X = Cr(left), Al(right)}.

Single crystal (Table 2) and powder X-ray diffraction analysis (Figure 3) revealed that for lighter lanthanides (till Ln = Ho), Series I was observed. These solids crystallize in the space group *Pca*2_*1*_; the cell parameters and the intensities of the reflections (Table 1) are comparable and in order with decreasing size of the respective ions. Of these, the compounds TbCr9, DyC10, HoCr11, LuCr29, PrAl18, NdAl19, TbAl23, DyAl24, HoAl25, ErAl26, TmAl27, and LuAl30 are being reported for the first time. The Series II compounds ErAl26 and TmAl27 exhibited different cell parameters when loaded at low temperatures (100K). A similar observation of phase transformation in Series II type solid has been reported in the case of TmCr12 (Zhang et al., 2008). In Series I, the cluster {X(OH)_6_Mo_6_O_18_}^3−^ with X = Al or Cr is the basic building block and coordinates with {Ln(H_2_O)_7_}^3+^ to form 1D zig-zag chains (Figure S1A). The solids are further stabilized via nonbonding interactions with lattice water (Figure S1B). The bond lengths and angles are comparable to other members already known (Fedoseev et al., 2002; Shivaiah et al., 2002, 2014; Gavrilova and Molchanov, 2005; Zhou and Yang, 2007; Cao et al., 2008; Shi D. M. et al., 2008; Zhao et al., 2008; Kumar et al., 2014a). An interesting observation is that under our reaction condition yttrium analogs crystallized as Series I (Table 1). It is not surprising since size of Y^3+^ in 9 CN (1.075 Å) is comparable to 1.083 Å of Dy^3+^ (Shannon, 1976; Lundberg et al., 2010). However, an earlier report had shown yttrium to have crystallized in Series II (Wang et al., 2011). It is clear from Figure 3 that a transition from Series I to II, under our experimental conditions, occurs around Erbium(Er). In the case of Er, reflections clearly indicate the presence of both Series I and II. Tm also shows a concomitant mixture of the two series. However, Yb appears to be predominantly that of Series II. We strongly believe that the presence of a second phase is quite likely in all higher lanthanides and this observation is only reflected in powder XRD (Figure 3).

**Table 1:** Refined cell parameters for solids in series I and series II.

**Ln/X**	**Ionic radius (Ln^**3+**^) in 9 and 8 CN (Å)**	**Series I: [Ln(H**_****2****_**O)**_****7****_**{X(OH)**_****6****_**Mo**_****6****_**O**_****18****_**}]·4H**_****2****_**O space group** ***Pca*****2**_****1****_			
		***a* (Å)**	***b* (Å)**	***c* (Å)**	**Volume (Å^**3**^)**
YCr1^#^	1.075^a^	10.889(6)	11.768(8)	22.263(13)	2853(5)
YAl1^#^	1.019	11.7567(8)	10.8939(7)	22.2145(15)	2845.2(3)
LaCr2	1.216^a^	11.831(4)	10.968(4)	22.621(8)	2935(3)
LaAl16	1.160	11.844(2)	11.010(2)	22.643(5)	2952.7
CeCr3	1.196^a^	11.777(6)	10.924(6)	22.549(11)	2901(4)
CeAl17	1.143	11.8255(15)	11.0007(14)	22.556(3)	2934.28
PrCr4	1.179^a^	11.771(6)	10.925(5)	22.442(12)	2886(4)
PrAl18	1.126	11.8173(6)	10.9707(5)	22.5088(11)	2918.1(2)
NdCr5	1.163^a^	11.762(5)	10.900(5)	22.425(9)	2875(3)
NdAl19	1.109	11.7659(5)	10.9321(5)	22.4151(10)	2883.2(2)
SmCr6	1.132^a^	11.759(4)	10.921(4)	22.372(8)	2873(3)
SmAl20	1.079	11.7700(6)	10.9257(6)	22.3586(12)	2875.2(3)
EuCr7	1.120^a^	11.778(10)	10.961(10)	22.422(19)	2895(4)
EuAl21	1.066	11.732(4)	10.909(3)	22.251(7)	2848(2)
GdCr8	1.107^a^	11.785(3)	10.926(3)	22.349(6)	2878(2)
GdAl22	1.053	11.716(13)	10.870(12)	22.23(3)	2836(9)
TbCr9	1.095^a^	11.8213(7)	10.9479(6)	22.3810(13)	2896.5(3)
TbAl23	1.040	11.7860(13)	10.9560(11)	22.333(2)	2883.9(5)
DyCr10	1.083^a^	11.7813(7)	10.9100(6)	22.2821(13)	2864.0(3)
DyAl24	1.027	11.7385(5)	10.8989(5)	22.2203(10)	2842.8(2)
HoCr11	1.072^a^	11.7696(10)	10.8829(9)	22.2283(18)	2847.2(4)
HoAl25	1.015	11.7114(11)	10.8538(10)	22.139(2)	2814.2(5)
ErCr12^#^	1.062^a^	11.7799(8)	10.8875(7)	22.2105(14)	2848.6(3)
ErAl26^#^	1.004	11.667(18)	10.846(18)	22.11(4)	2797(8)
TmCr13^#^	1.052^a^ 0.994	11.7665(8)	10.8728(8)	22.2149(16)	2842.1(4)
**Ln/X**	**Series II: [Ln(H**_**2**_**O)**_**7**_**{X(OH)**_**6**_**Mo**_**6**_**O**_**18**_**}Ln(H**_**2**_**O)**_**7**_**]{X(OH)**_**6**_**Mo**_**6**_**O**_**18**_**}·16H**_**2**_**O space group** ***P*** $\bar{1}$						
	***a*** **(Å)**	***b*** **(Å)**	***c*** **(Å)**	**α (°)**	**β (°)**	**γ (°)**	**Volume (Å**^**3**^**)**
ErCr12	10.9762(6)	11.6149(7)	13.9397(8)	74.4390(10)	83.8050(10)	89.4570(10)	1701.65(17)
ErAl26	11.038(19)	11.067(3)	14.01(4)	74.67(10)	84.10(8)	89.41(7)	1731(10)
ErAl26*	15.5455(9)	15.8776(9)	16.0412(9)	86.9390(10)	72.4000(10)	64.5440(10)	3394.1(3)
TmCr13	11.0989(13)	11.7203(14)	13.9843(40)	74.993(2)	84.528(2)	89.545(2)	1748.9
TmAl27	10.9836(4)	11.5857(4)	13.9171(5)	74.4310(10)	83.8200(10)	89.4490(10)	1695.75(11)
TmAl27*	15.5445(12)	15.8913(12)	16.0467(12)	86.9470(10)	72.8530(10)	64.5140(10)	3396.1(4)
YbCr14	11.0379(5)	11.6436(5)	13.9044(7)	75.005(1)	84.530(1)	89.466(1)	1718.08(14)
YbAl28	11.011(10)	11.585(12)	13.885(13)	74.895(19)	84.51(2)	89.517(18)	1702(3)
YCr1	11.0492(6)	11.6488(6)	13.9350(8)	75.070(4)	84.551(4)	89.536(4)	1724.94(16)
YAl15	11.052(5)	11.620(5)	13.947(5)	75.006(5)	84.535(5)	89.582(5)	1722.0(12)

a Ionic radii of Ln^3+^ in coordination number 9. Rest are for coordination number 8. The values are calculated from r^3^ V plots.

# *Represents the solids of series I wherein the solids of series II are also present as a concomitant phase*.

**Table 2:** Crystal data and refinement details for all single crystals reported for the first time.

**Parameter**	**TbCr9**	**DyCr10**	**HoCr11**	**ErCr12^#^**	**TmCr13^#^**	**LuCr29**	**PrAl18**	**NdAl19**	**TbAl23**
Formula	Cr_2_Mo_12_O_70_Tb_2_	Cr_2_Dy_2_Mo_12_O_70_	Cr_2_Ho_2_Mo_12_O_70_	Cr_2_Er_2_Mo_12_O_70_	Cr_2_Mo_12_O_70_Tm_2_	Cr_2_Mo_12_O_78_ Lu_2_	Al_2_Mo_12_O_70_Pr_2_	Al_2_Mo_12_Nd_2_O_70_	Al_2_Mo_12_O_70_Tb_2_
Formula weight	2,693.14	2,732.28	2,705.14	2,741.80	2,713.14	2,853.22	2,607.06	2,613.72	2,643.10
T (K)	298(2)	100(2)	100(2)	100(2)	100(2)	298(2)	298(2)	100(2)	298(2)
Crystal system	Orthorhombic	Orthorhombic	Orthorhombic	Orthorhombic	Orthorhombic	Triclinic	Orthorhombic	Orthorhombic	Orthorhombic
Space group	*Pca*2_1_	*Pca*2_1_	*Pca*2_1_	*Pca*2_1_	*Pca*2_1_	*P $\bar{1}$*	*Pca*2_1_	*Pca*2_1_	*Pca*2_1_
*a* (Å)	11.8213(7)	12.0050(7)	11.8069(12)	11.7799(8)	11.7665(8)	11.0470(2)	11.8173(6)	11.7210(6)	11.7860(13)
*b* (Å)	10.9479(6)	11.0090(6)	10.9263(11)	10.8875(7)	10.8728(8)	11.6690(2)	10.9707(5)	10.8990(5)	10.9560(11)
*c* (Å)	22.3810(13)	22.660(13)	22.2930(2)	22.2105(14)	22.2149(16)	13.9150(3)	22.5088(11)	22.3370(11)	22.3330(2)
α (°)	90	90	90	90	90	74.937(4)	90	90	90
β (°)	90	90	90	90	90	11.669(2)	90	90	90
γ (°)	90	90	90	90	90	13.915(3)	90	90	90
V (Å^3^)	2,896.5(3)	2,864.0(3)	2,875.9(5)	2,848.6(3)	2,842.1(4)	1,724.0(6)	2,918.1(2)	2,853.2(2)	2,883.9(5)
Z	2	2	2	2	2	1	2	2	2
Dcalc (gcm^−3^)	3.086	3.006	3.161	3.197	3.170	2.748	2.967	3.042	3.044
μ_MoKα_ (cm^−1^)	5.402	5.320	5.737	5.961	6.139	5.365	4.286	4.494	5.100
Theta range (°)	2.60–31.31	2.50–35.50	2.54–24.57	2.55, 26.44	2.55–28.54	2.51–30.75	2.53–30.44	2.62–31.30	2.54–31.05
R_1_, wR_2_ [I >2σ(I)]^a^	0.0484, 0.1236	0.0744, 0.1985	0.0623, 0.1364	0.0799, 0.1603	0.0695, 0.1964	0.0510, 0.1477	0.0309, 0.0901	0.0442, 0.1051	0.0606, 0.1488
GOF	1.022	1.539	1.064	1.164	1.619	1.275	0.786	0.853	1.088
ICSD no.	1881544	1821829	1881881	1881880	1881833	1881617	1881543	1881610	1881555
**Parameter**	**DyAl24**	**HoAl25**	**ErAl26^#^**	ErAl26*	**TmAl27**	TmAl27*	**LuAl30**
Formula	Al_2_Dy_2_Mo_12_O_70_	Al_2_Ho_2_Mo_12_O_70_	Al_2_Mo_12_ Er_2_O_70_	Al_2_Er_2_Mo_12_O_78_	Al_2_Mo_12_O_78_Tm_2_	Al_2_Mo_12_Tm_2_O_78_	Al_2_Mo_12_Lu_2_O_78_
Formula weight	2,650.24	2,687.10	2,693.14	2,803.76	2,803.20	2,791.10	2,803.18
T (K)	100(2)	100(2)	100(2)	100(2)	100(2)	100(2)	298(2)
Crystal system	Orthorhombic	Orthorhombic	Orthorhombic	Triclinic	Triclinic	Triclinic	Triclinic
Space group	*Pca*2_1_	*Pca*2_1_	*Pca*2_1_	*P $\bar{1}$*	*P $\bar{1}$*	*P $\bar{1}$*	*P $\bar{1}$*
*a* (Å)	11.7385(5)	11.7114(11)	11.667(18)	15.5455(9)	11.0276(7)	15.5445(12)	11.0453(14)
*b* (Å)	10.8989(5)	10.8538(10)	10.846(18)	15.8776(9)	11.5944(7)	15.8913(12)	11.6203(14)
*c* (Å)	22.2203(10)	22.1390(2)	22.110(4)	16.0412(9)	13.8969(9)	16.0467(12)	13.8854(17)
α (°)	90	90	90	86.9390(10)	74.981(2)	86.947(1)	74.975(2)
β (°)	90	90	90	72.4000(10)	84.559(2)	72.358(1)	84.571(2)
γ (°)	90	90	90	64.5440(10)	89.539(2)	64.514(1)	89.568(2)
V (Å^3^)	2,842.8(2)	2,814.2(4)	2,797.0(8)	3,394.1(3)	1,708.15(19)	3,396.1(4)	1,713.3(4)
Z	2	2	2	2	1	2	1
Dcalc (gcm^−3^)	3.096	3.133	3.157	2.740	2.725	2.730	2.717
μ_MoKα_ (cm^−1^)	5.314	5.524	5.728	4.738	4.847	4.876	5.124
Theta range (°)	2.55–30.74	2.56–20.92	2.55–27.27	2.28–30.75	2.29–29.09	2.58–30.92	2.51–30.86
R_1_, wR_2_ [I >2σ(I)]^a^	0.0308, 0.0765	0.0571, 0.1415	0.1078, 0.1693	0.0435, 0.1086	0.0557, 0.1000	0.0787, 0.3366	0.0310, 0.1056
GOF	1.020	1.055	1.196	1.027	1.047	2.413	0.861
ICSD no.	1881556	1881882	1881830	1881609	1881832	1881834	1881831

# *Represents the solids of series I wherein the solids of series II are also present as a concomitant phase*.

Crystallization reaction of Anderson-Evans cluster with heavier lanthanides (Er to Yb) led to Series II. These solids crystallized in *P* $\bar{1}$ space group (Table 2). Though the stoichiometry of lanthanide to the cluster in the crystal structure remained the same as in Series I, the assembly of the molecular solid (0D) was different. Also, the structure contained a larger number of uncoordinated water molecules. In Series II, the cationic cluster [Ln(H_2_O)_7_{X(OH)_6_Mo_6_O_18_}Ln(H_2_O)_7_]^3+^ derivatised by a pair of lanthanide hydrates involved in complex H-bonding with another discrete anionic cluster {X(OH)_6_Mo_6_O_18_}^3−^ (Figure S2). Two groups have crystallized and structurally characterized a few members belonging to Series II (Zhang et al., 2008; Wang et al., 2011). The previous works as well the present one clearly suggest that a few lattice water are disordered in solids belonging to Series II. Bond valence sums conclusively show that lanthanide, chromium/aluminum and molybdenum occur in +3, +3, and +6 oxidation state, respectively.

**Figure 4:**
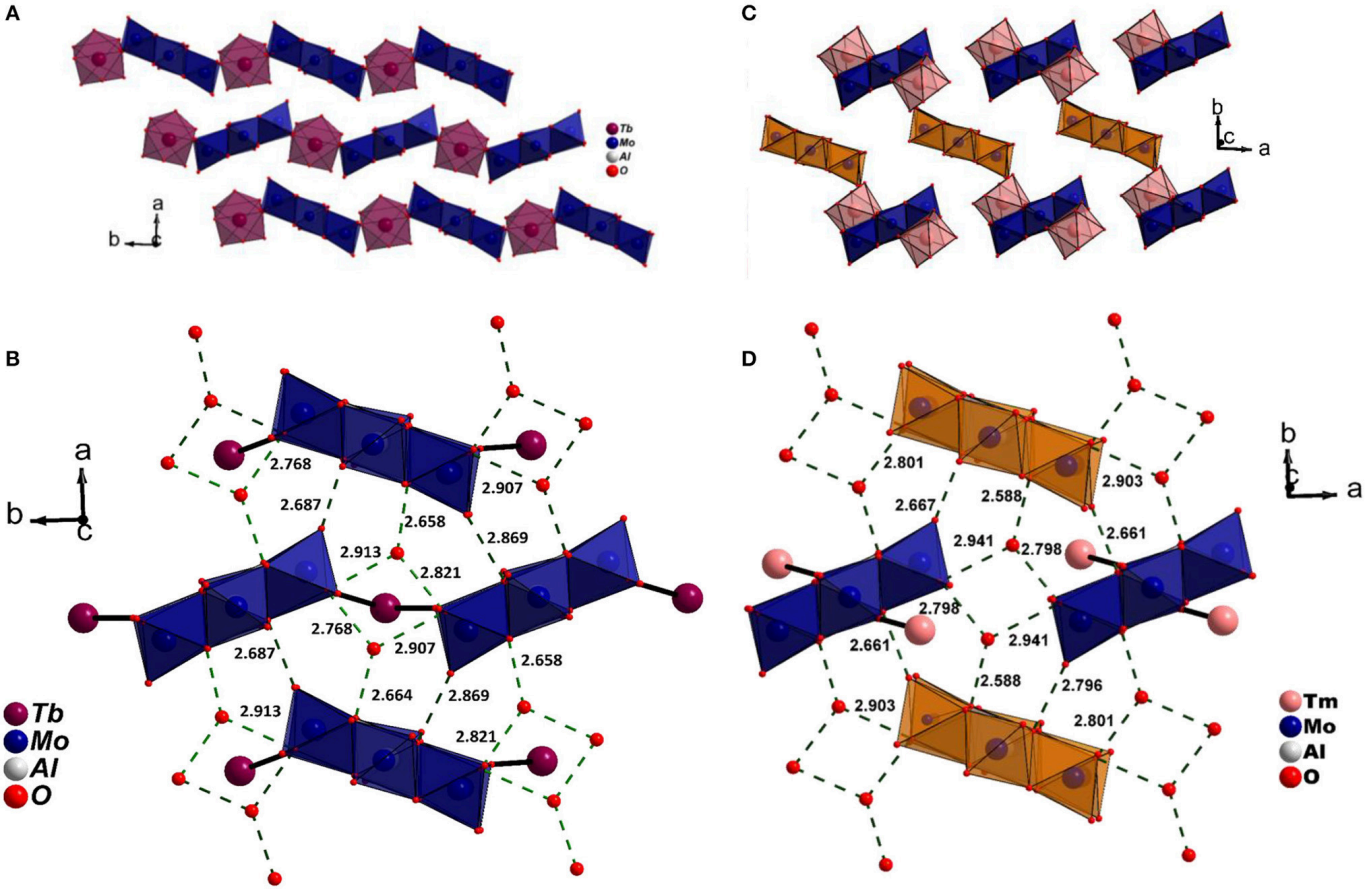
**(A)** Stacking of 1D zig-zag chains on *ab* plane and **(B)** 1D chains stabilized through O_Mo_-H···Ow in Series I. **(C)** Growth of 0D molecular solid on *ab*plane. Apart from electrostatic interaction, the lanthanide complex derivatised by the cluster, [{Tm(H_2_O)_7_{Al(OH)_6_Mo_6_O_18_}Tm(H_2_O)_7_}]^3+^ and the anionic cluster, {Al(OH)_6_Mo_6_O_18_} also interact through O_Mo_-H···Ow. Notice the strikingly similar H-bonding pattern on *ab* plane in **(B)** Series I and **(D)** Series II. In **(C,D)** the anionic clusters are depicted in orange. The water molecules coordinated to lanthanide cations are omitted for clarity.

**Figure 5:**
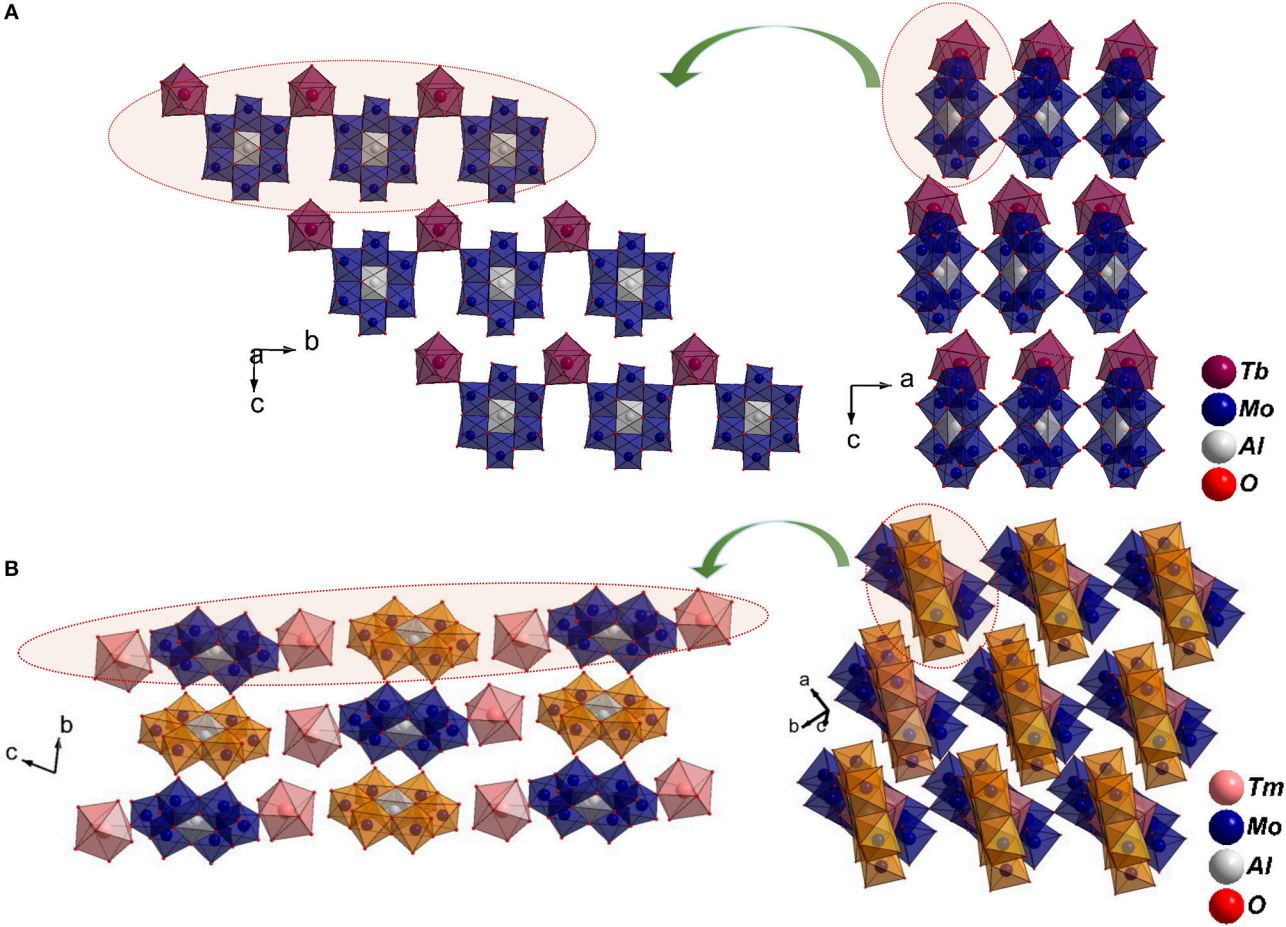
**(A)** 1D chains along [010] and its projection on *bc* plane in series I. **(B)** The discontinuous arrangement of cations and anions in series II stacked one over the other closely resemble series I. The discrete anionic Anderson-Evans cluster is depicted in orange for clarity.

A close inspection of the structures, however revealed that the crystal packing of the complex cation, and the discrete anion, showed a strong resemblance to Series I (Figures 4A,C). It is important to point out that the non-bonding interactions of the two series on the *ab* plane are strikingly similar (Figures 4B,D) signifying the small energy difference between the two supramolecular aggregates. However, since higher lanthanides favor a lower coordination number due to smaller size, an extended chain appears to be unfavorable. Interestingly, Ln^3+^ coordinates to terminal oxygen (O_t_) of the hexagonal cluster at 1,4 positions (Series II) in contrast to 1,3 found in Series I (Figures S1, S2). An efficient close packing probably necessitates the formation of a complex cation wherein the cluster is coordinated by a pair of [Ln(H_2_O)_7_]^3+^ via symmetry equivalent terminal oxygen atoms. As a consequence, the complex cations and anions stack one over the other in a twisted fashion (Figure 5). The packing is further facilitated by non-bonding interactions with additional water molecules that get incorporated into the structure. The present study as well as the two literature reports clearly show that the occurrence of a significant disorder among lattice water thus making it difficult to obtain a reliable model.

#### Crystallization of Lanthanide Coordinated Chromium/Aluminum Molybdates

A molybdenum source dissolved in aqueous medium under varying concentration and *p*H may contain numerous soluble molybdate species (Tkac and Paulenova, 2008). Under our experimental conditions, it is reasonable to assume the occurrence of stable Anderson-Evans cluster (Pavani et al., 2011). Crystallization is a supramolecular reaction (Desiraju, 2007; Singh et al., 2010a; Desiraju et al., 2011; Singh and Ramanan, 2011) and hence a crystal separating from a medium can be considered to occur from the aggregation of appropriate molecular species including solvent molecules. In the context of crystal engineering of lanthanide coordinated with chromium or aluminum molybdate cluster, two species, *viz*. {Ln(H_2_O)_n_}^3+^ and {X(OH)_6_Mo_6_O_18_}^3−^ aggregate along with water molecules leading to a stable crystal, belonging to Series I or II (Singh et al., 2010b, 2014; Pavani et al., 2011; Singh and Ramanan, 2011; Kumar et al., 2014b). In Series I, the cations and anions condense forming extended chains through coordination linkages whereas in Series II, the smaller size of lanthanides results in the formation of a discrete cationic complex wherein the cluster is coordinated by a pair of lanthanide ions. The packing of the cations and the discrete cluster anions are facilitated through nonbonding interactions and hence more water molecules get incorporated into the structure. The two closely related isostructural series represent an intriguing example of crystal engineering of salt hydrates manifested by parsimonious nature to compromise between Kitaigorodskii's close packing principle (Kitaigorodskii, 1965) and non-covalent interactions assembling into a stable crystal under a given condition. The principle appears to be the same operating in a simple salt hydrate crystallizing from a supramolecular assembly of water-mediated {Cu(H_2_O)_6_}^2+^ and ${\text{SO}}_{4}{}^{2-}$ leading to 0D (molecular) CuSO_4_·7H_2_O (Boothite), 1D (chain) CuSO_4_·5H_2_O (Chalcanthite) and multidimensional CuSO_4_·3H_2_O(Bonattite), CuSO_4_·H_2_O (Poitevinte) and CuSO_4_ (Chalcocyanite) or organic hydrates (Upreti et al., 2007; Singh et al., 2010a).

### Excitation and Emission Spectra of Rare-Earth Chromium/Aluminum Molybdates

**Figure 6:**
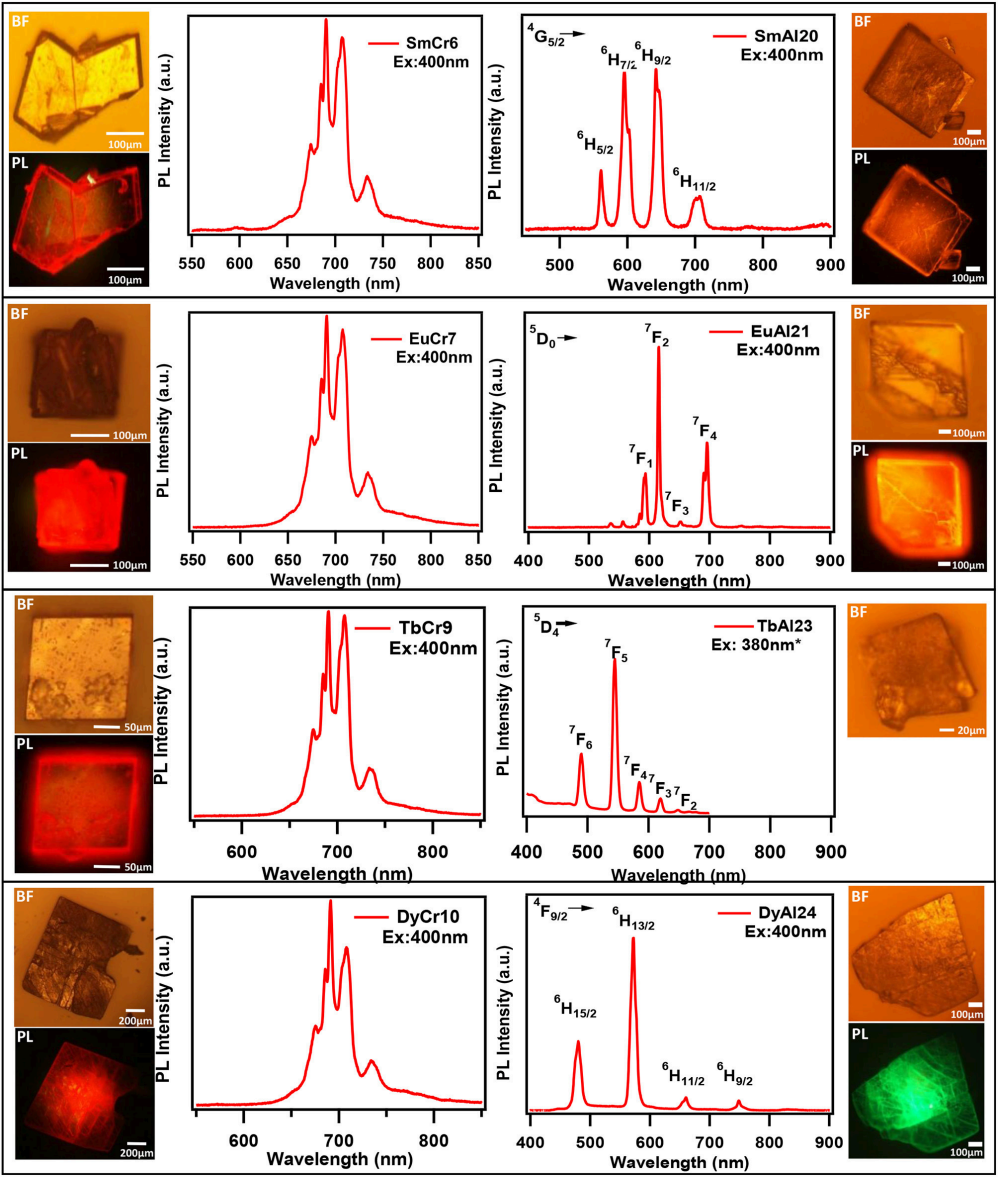
The Emission spectra of single crystals of **(A)** Ln(H_2_O)_7_{Cr(OH)_6_Mo_6_O_18_}]·yH_2_O and **(B)** [Ln(H_2_O)_7_{Al(OH)_6_Mo_6_O_18_}]·yH_2_O. Ln = Sm, Eu, Tb, Dy, and Tm. The Bright field (BF) and PL images (PL) are given at left and right sides for respective crystals. PL spectra and images are recorded using 400 nm laser excitation coupled to a microscope. *Excited by 380 nm of Xe lamp coupled to a monochromator (see text).

**Figure 7:**
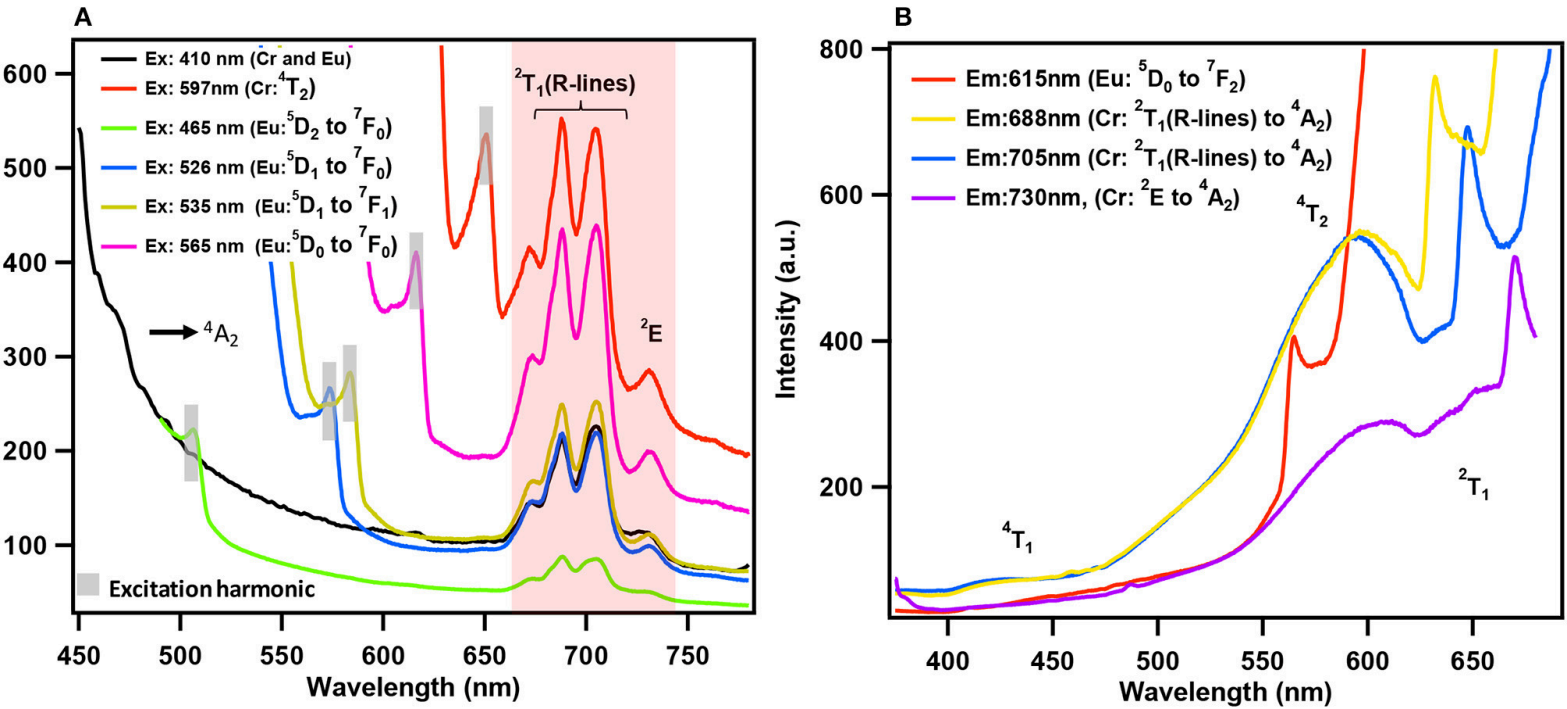
**(A)** Emission spectra of EuCr7 solid, excited at various absorption energy levels of Eu^3+^ and Cr^3+^ ions. **(B)** Excitation spectra of EuCr7 solid, monitored at various expected emission energy levels of Eu^3+^ and Cr^3+^ ions. The emission spectra are dominated by Cr^3+^ emission, even though the excitation was at different Eu^3+^ energy levels, confirms the domination of Cr^3+^ emission with suggestion of possible energy transfer from the excited levels of Eu^3+^ to Cr^3+^ (see text).

Till recently there are only five papers that have reported emission (PL) of solids containing lanthanides and chromium molybdate cluster (Yusov et al., 2002; Cao et al., 2008; Shi D. M. et al., 2008; Yang et al., 2013; Kumar et al., 2014a). Generally, polycrystalline samples may be contaminated and are prone to react with air. In this work, we carried out PL studies directly on single crystals. Microscopic PL spectra and images are obtained from a modified microscope. Both excitation (PLE) and emission (PL) studies were conducted on commercial spectrofluorophotometer (see Experimental section). As visible to naked eye, all rare-earth containing chromium molybdate crystals (Ln = La, Ce, Pr, Nd, Sm, Eu, Gd, Tb, Dy, Ho, Er, Tm, Yb, and Y) exhibit strong reddish-purple emission. In Figure 6, we have shown emission spectra of all the rare-earth containing chromium molybdate solids (refer also Figure S10). This is to be noted that only Sm, Eu, Tb, and Dy are known to show strong emission in the visible region. The corresponding aluminum analogs (Figure 6) showed only characteristic emission from the respective lanthanide ions. In general, lanthanides are prone to give strong discrete emission due to *f-f* transitions, whereas Cr^3+^ is from 3*d* band contribution. The domination of Cr^3+^ emission from the lanthanide containing chromium molybdates still remains an intriguing problem. Europium doped solids (crystalline phosphors and glasses) strongly emitting in orange-red region are known structural probes for identification of symmetry and coordination (Swapna et al., 2014). To analyze further, we recorded PL spectra of EuCr7 at various excitation levels of both Cr^3+^ and Eu^3+^ transitions (Figure 7). For all excitation energies, we observed only signatures of Cr^3+^ ion, occurring at around 692, 709 and 733 nm attributed to Stokes shifted ^2^T_1_→^4^A_2_ (R-lines) and ^2^E → ^4^A_2_ transitions. It is widely accepted that Cr^3+^ ion emission is highly dependent on temperature and on the matrix in which the ion is situated. The sharp emission around 703 and 704.4 nm observed for Cr^3+^ in [Na_3_{Cr(OH)_6_Mo_6_O_18_]·8H_2_O at 4.2 K was assigned to R-lines (Yamase and Sugeta, 1993). Similar to our earlier observation (Kumar et al., 2014a), the room-temperature spectra of all chromium molybdates showed only the dominant red emission (Figure 6). The purple color strikingly visible to naked eye suggests that non-radiative processes leading to low emission quantum yield of R-lines is unlikely.

**Figure 8:**
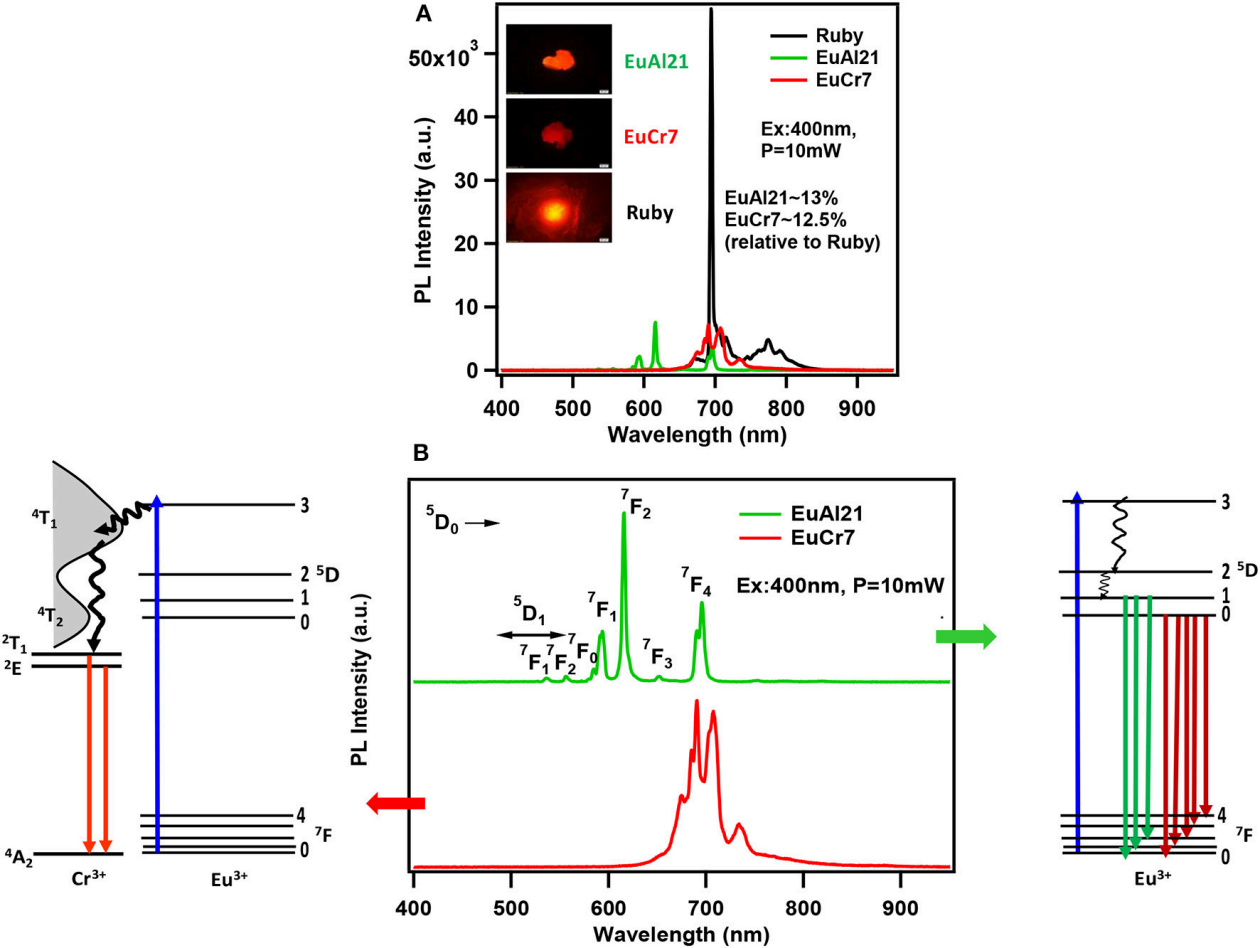
**(A)** The relative emission spectra comparison between EuAl21, EuCr7, and naturally occurring Ruby recorded under similar conditions (Excited by 400 nm diode laser, ~10 mW). Corresponding PL images are shown in the inset. **(B)** Emission spectra of EuAl21 and EuCr7 along with the schematic representation of emission mechanism for either energy transfer between lanthanide and lower lying Cr^3+^energy levels (EuCr7) or within the Eu^3+^ ion energy levels (EuAl21).

The excitation (PLE) spectra at the prominent emission lines of Cr^3+^ and Eu^3+^ ions (Figure 7) observed at 423, 581 and 648 nm correspond to absorption transitions of Cr^3+^ in pseudo-octahedral geometry, ^4^A_2_→^4^T_1_ (F), ^4^T_2_, and ^2^T_1_. Therefore, it is convenient to conclude that the observed emission in EuCr7 and other chromium analogs is essentially from Cr^3+^ without any traces of contribution from the optically active lanthanide ions. PLE and PL spectra shown in Figure 7, categorically confirms the domination of Cr^3+^ emission suggesting a possible energy transfer from excited levels of Eu^3+^ to Cr^3+^. Figure 8, shows a comparison of relative emission intensities of EuCr7 and EuAl21 single crystals, along with natural ruby crystal. The experiments are conducted under identical condition (same excitation power densities, integration time etc). The relative PL intensities and profile broadly suggest dominance of Cr^3+^ emission from EuCr7 and characteristic emission of Eu^3+^ from EuAl21. The relative intensities of both solids are about 13% on comparison with the natural ruby. Therefore, in EuCr7 it may be concluded that while excited by 400 nm (^5^D_3_ level of Eu^3+^) (or any other transitions of ^5^D_J_(*J* = 0, 1, 2) levels), the excited densities are completely energy transferred to the equivalent energy bands of ^4^T_1_ and ^4^T_2_, then depopulates radiatively from ^2^T_1_ and ^2^E to ground state ^4^A_2_ of Cr^3+^ ion. On the other hand, EuAl21 solid, the excitation to until ^5^D_0_ level, results into the emission from ^5^D_1_ to ^7^F_J_(*J* = 0, 1, 2) and ^5^D_0_ to ^7^F_J_(*J* = 0, 1, 2, 3, 4) of Eu^3+^ ion. The schematic energy levels of EuCr7 and EuAl21are given in Figure 8.

It is to be noted that the absence of Eu^3+^ emission and the presence of ruby-like emission (at 77 K) in [Eu(H_2_O)_7_{Cr(OH)_6_Mo_6_O_18_}] was also observed previously by another group (Yusov et al., 2002). Surprisingly, two solids reported in the literature have exhibited contrasting results. [Eu(H_2_O)_7_{Cr(OH)_6_Mo_6_O_18_}]·4H_2_O (Shi D. M. et al., 2008) showed only characteristic emission due to Eu^3+^ ions with no trace of Cr^3+^ emission. Similarly, the molecular solid [(C_6_H_5_NO_2_)_2_{Dy(H_2_O)_6_}{(Cr(OH)_6_Mo_6_O_18_)}]· 2(C_6_H_5_NO_2_)· 6H_2_O also showed characteristic emission only from Dy^3+^ ion (Yang et al., 2013). It can be argued that in the latter solid, there is no direct coordination linkage between the lanthanide and the cluster. However, another molecular solid, [(Hbipy)_2_{(Cr(OH)_6_Mo_6_O_17_(OH)}] displayed typical Cr^3+^ emission only (Li et al., 2016). Therefore, having the extensive experimental evidence from the present study, the suppression of lanthanide emission and dominant chromium emission could be attributed to strong absorption cross-section of Cr^3+^ ions and energy transfer (ET) between 4f−4f emission transitions of Ln^3+^ ions to that of Cr^3+^*d*-orbital energy levels. While all the compounds showed bright red emission, in the special case of NdCr5, the red emission from Cr^3+^ is considerably quenched. Figure S8 shows a comparison of the same with EuCr7, recorded under similar condition, along with PL images. It can be speculated that Cr^3+^emitting energy level ^2^E is closer to Nd^3+^ level ^4^F_3/2_, therefore the quenching of emission intensity may be possible (Weber, 1973); However, further studies are required to address this issue.

One of the characteristic features of a trivalent lanthanide ion is the strong and sharp 4f shell absorption and emission peaks that cover a wide range of spectral regions, from UV to IR. It is known that the lanthanide 4f−4f transition strengths and positions are sensitive to the local environment within a crystalline network. PL studies of the aluminum analogs (Figure 6) revealed that the solids containing Sm, Eu, and Dy ions exhibited characteristic emission as expected (de Bettencourt-Dias, 2014). In Table 3, we have also shown the excitation and emission spectra with corresponding assignments. The visible emitting TbAl23 excitation and emission spectra are given in Table 3 and Figure S7. Since Tb^3+^ does not have any energy level corresponding to the diode laser excitation source (400 nm), the microscopic PL spectra and images could not be obtained in Figure 6. The aluminum analogs of Ce^3+^, Pr^3+^, Ho^3+^, and Er^3+^ compounds did not show any appreciable PL in the visible region while excited in the UV- blue region. Another UV-violet low emitting solid TmAl27 and Cr^3+^ emission dominated TmCr13 PL spectra are shown in Figure S8.

**Table 3:** Excitation and emission spectra of optically active lanthanides along with corresponding energy levels of Sm, Eu, Tb, and Dy. All the spectra are taken by a spectrofluorophotometer utilizing xenon lamp (see text). All assigned transitions (in parenthesis) are as per the reference (de Bettencourt-Dias, 2014).

**Ln^**+3**^**	**Emission transitions Experimental (Literature value)**			**Excitation and emission spectra**
	**Excited state**	**Ground state**	**Wavelength (nm)**
Sm	^4^G_5/2_	^6^H_5/2_^6^H_7/2_^6^H_9/2_^6^H_11/2_	560 (560) 595 (595) 642 (640) 720 (700)	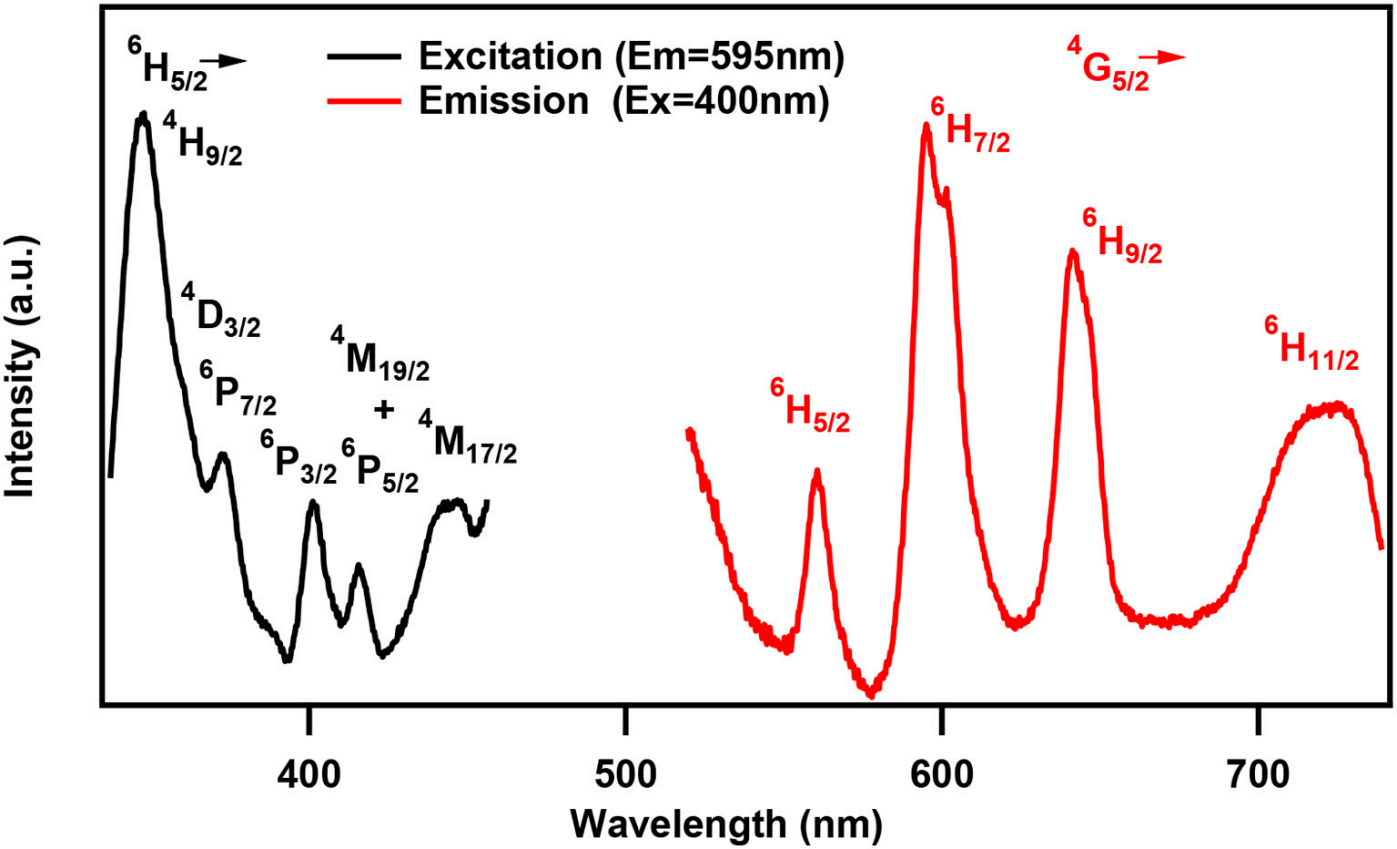
Eu	^5^D_1_^5^D_0_	^7^F_0_^7^F_1_^7^F_2_^7^F_0_^7^F_1_^7^F_2_^7^F_3_^7^F_4_	525 (525) 535 (530) 555 (560) 583 (583) 592 (590) 615 (615) 650 (650) 694 (720)	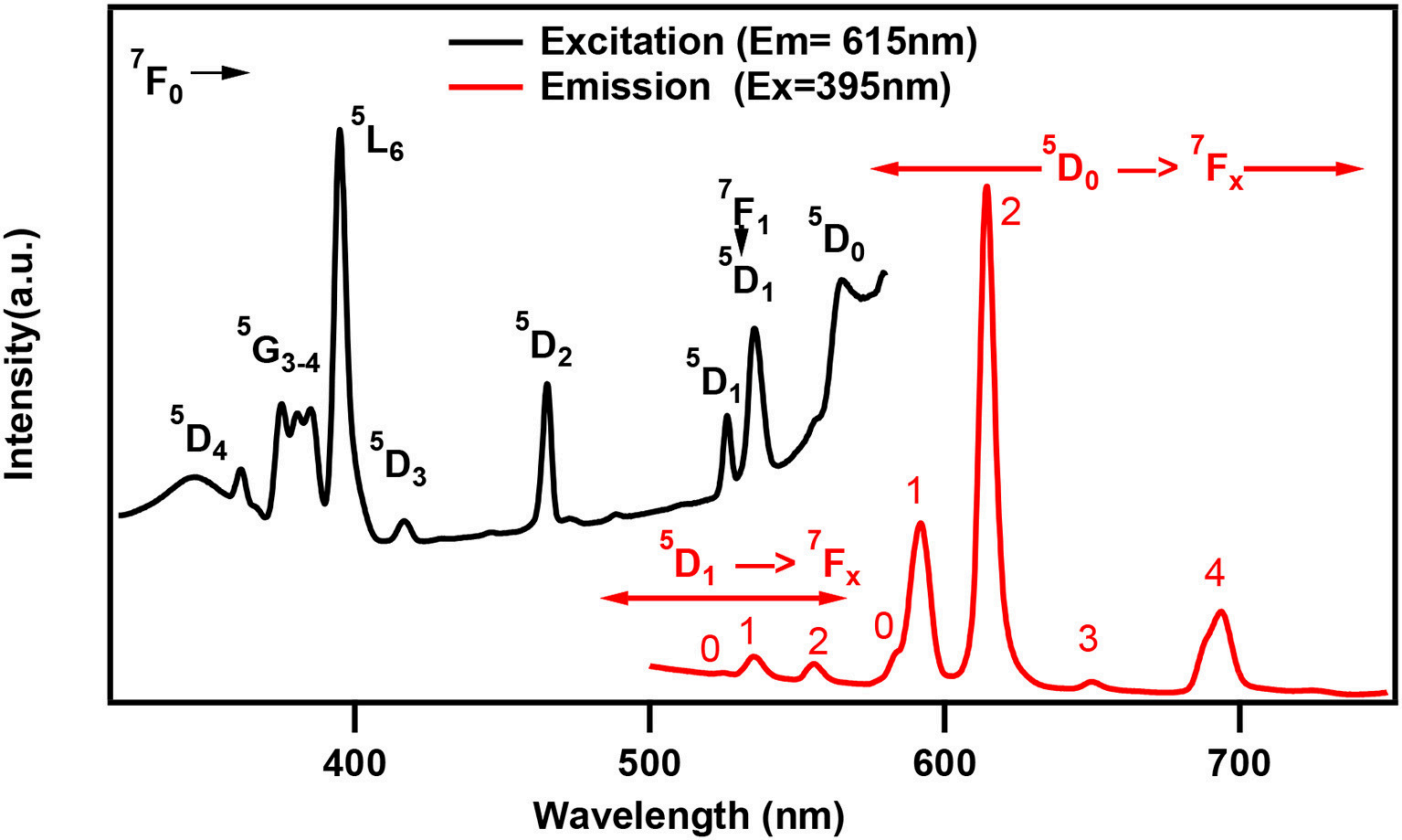
Tb	^5^D_4_	^7^F_6_^7^F_5_^7^F_4_^7^F_3_^7^F_2_	490 (490) 545 (540) 585 (580) 620 (620) 648 (650)	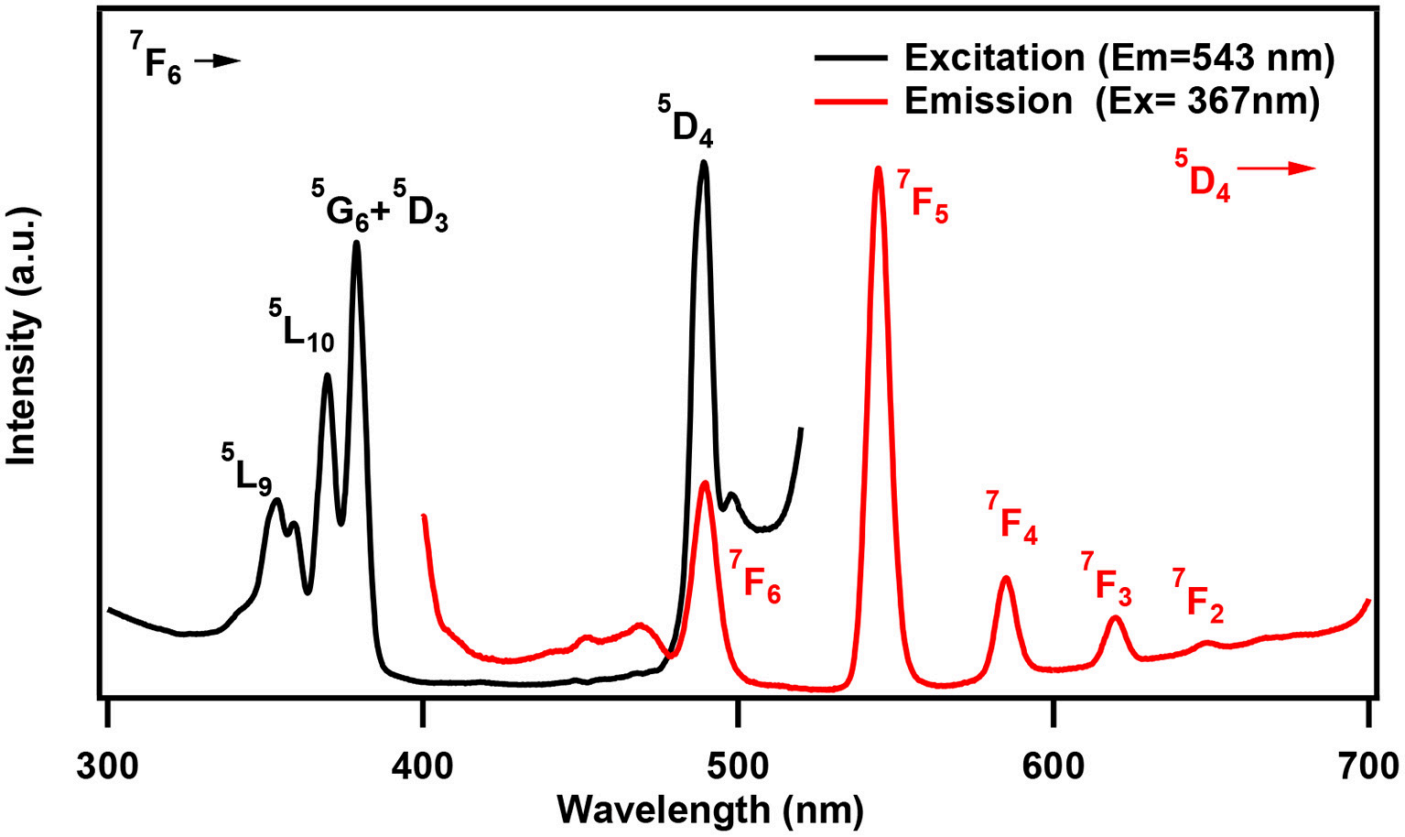
Dy	^4^F_9/2_	^6^H_15/2_^6^H_13/2_^6^H_11/2_^6^H_9/2_	480 (475) 572 (570) 658 (660) 721 (750)	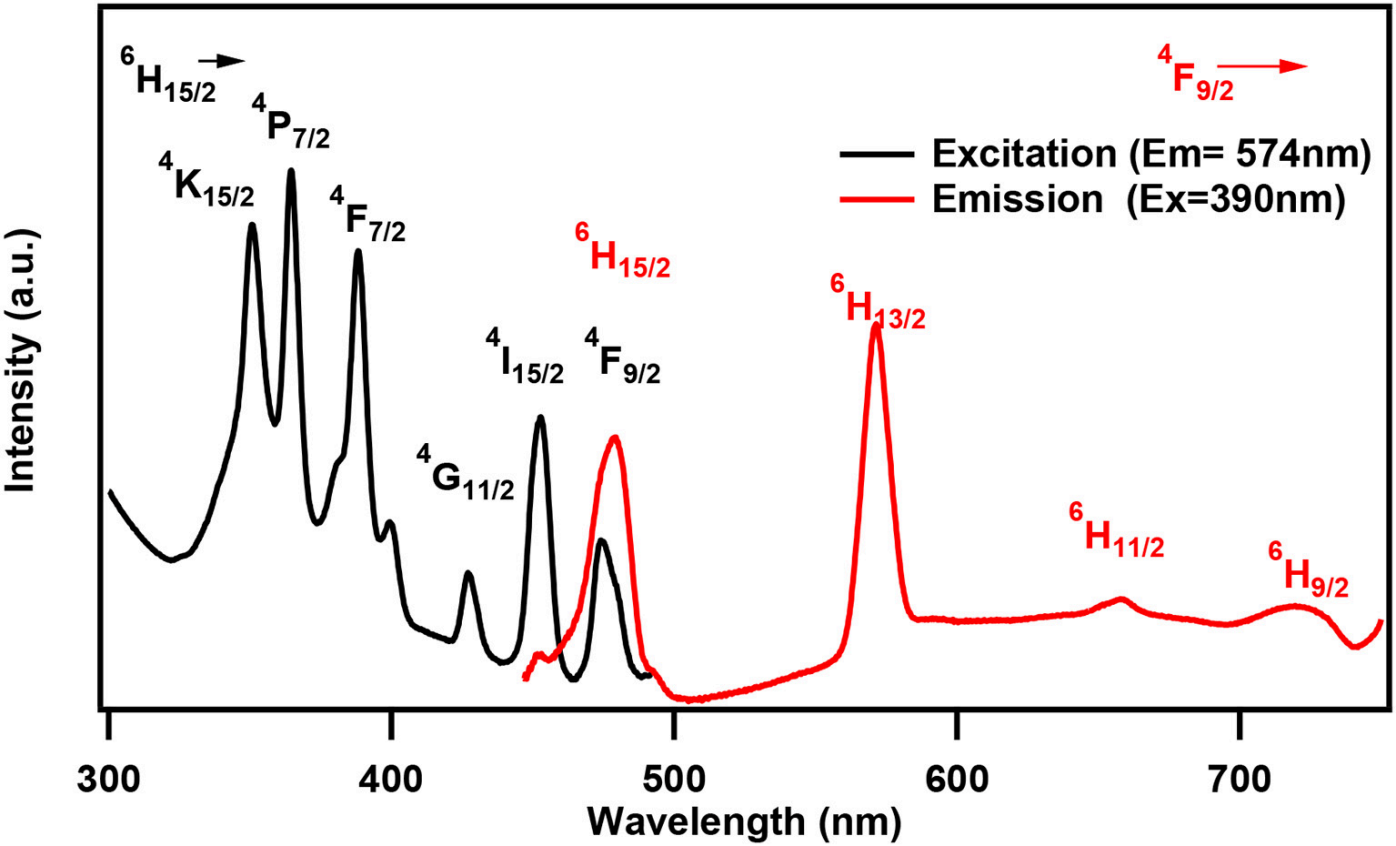

## Physical Measurements

### Single-Crystal X-ray Diffraction

Data collections were performed on a Bruker D8-Quest diffractometer with a Turbo X-ray Source (MoKα radiation, λ = 0.71073 Å) adopting the direct drive rotating anode technique and a PHOTON detector. The data frames were collected using the program APEX3 and processed using the program SAINT routine in APEX3. The structures were solved by direct methods and refined by the full-matrix least-squares on *F*^2^ using the SHELXTL-2014 program.

### Powder X-ray Diffraction

Room temperature powder X-ray diffraction data (PXRD) were collected on a Bruker D8 Advance diffractometer using Ni-filtered CuKα radiation employing a step size of 0.02 and at count time of 1 s per step over the range 5° < 2θ < 80°. Rietveld refinement of powder diffraction data of all polycrystalline samples were carried out using Topas 4.2, Bruker for ensuring homogeneity of the synthesized products (Table S1).

### Optical Measurements

Visible emission (~400–700 nm) and excitation spectra (300–520 nm) were recorded using a Spectrofluorophotometer (Shimadzu RF-5301PC), where the Xenon lamp is used as light source (Figure 6 and Table 3). The microscopic optical images (Bright field), PL images and visible emission spectra were recorded using a modified confocal high-resolution microscope (Olympus BX-51) (Figures 6–8). The modified microscope is equipped with a fiber coupled 400 nm (±5 nm) diode laser and xenon lamp as sources. The emission spectra and images were collected, respectively by a fiber optic spectrometer (Ocean Optics, Maya 2000Pro) and a camera (Olympus, DP26) through a 425 nm long-pass filter (Pradeesh et al., 2009; Optics Express) in specular reflection mode.

### Vibrational Spectroscopy

FT-IR was recorded on a Nicolet 5DX spectrophotometer with pressed KBr pellets in the range of 4,000–400 cm^−1^. All the peaks are consistent with literature reports (Fedoseev et al., 2002; Bridgeman, 2006; Kumar et al., 2014a) (Figures S2A–X, S3A–D). The broad peaks at 3,500–3,200 cm^−1^ correspond the O–H bond stretch. Due to differently bound water molecules, there is an observable splitting of these bands. The peaks at 1,630–1,620 cm^−1^ are characteristic of the deformation vibrations of H–O–H owing to the presence of coordinated and lattice water molecules. A peak at about 1,400 cm^−1^ in the case of Pr is observed, which has been reported by another group, which could not been explained adequately (Fedoseev et al., 2002). The low wavenumber peaks (960–400 cm^−1^) are alike and are characteristic of the Anderson-Evans cluster. The bands at 950–890 cm^−1^ correspond to the symmetric stretching frequencies of the Mo–O_t_ bonds. The shape and intensity of the peaks are not sensitive to the nature of both the lanthanide and the cluster. The bands due to the vibrations of the bridging Mo–O–Mo bonds of different types are observed in the 700–400 cm^−1^region. The replacement of the larger Cr^3+^ ion by Al^3+^ results in a noticeable shift of the vibrations of Mo–O–Mo from ~460–440 in case of Al to ~410–420 cm^−1^ in case of Cr.

### Thermal Studies

Thermogravimetric analysis for selected solids were carried out using a Perkin-Elmer TGA7 system on well ground samples under a flowing nitrogen atmosphere with a heating rate of 10°C min^−1^ in the range 40–800°C. The weight loss is almost consistent throughout the scan making it difficult to assign loss in steps. The presence of concomitant phases in the heavier lanthanides (Er and Tm) also makes it difficult to assign weight loss appropriately (Figures S3A–H).

As expected, thermal behavior of the two series (I and II) of solids were slightly different. For series I, the first weight loss (11–13%) occurred in the range of 40–120°C corresponding to the four lattice and two coordinated water. The slope of the curve clearly suggests the instability of an expected intermediate phase, [Ln(H_2_O)_7_{X(OH)_6_Mo_6_O_18_}]. The second weight loss of about 5–6% in the range of 150–330°C possibly corresponds to the loss of remaining coordinated water molecules. The total weight loss ~18–20% till ~500°C was in good agreement with complete decomposition of the solids to the respective oxides including the loss of hydroxyl groups attached to the cluster. For series II, the first weight loss (40–170°C) of about 10–11% corresponds to the loss of sixteen water molecules while the second one (170–320°C) about 9–10% correspond to the loss of fourteen coordinated water molecules. It is to be noted that for Er and Tm, the total weight loss showed significant deviation. This can be ascribed to the presence of a mixture of series I and II. Our TGA results are comparable to the earlier reports (Cao et al., 2008; Shi D. M. et al., 2008; Zhang et al., 2008; Wang et al., 2011).

## Conclusions

Our strategy to assemble Anderson-Evans cluster and lanthanide hydrate through a one-pot synthesis led to two closely related structures of the composition [Ln(H_2_O)_7_{X(OH)_6_Mo_6_O_18_}]·yH_2_O (X = Cr or Al). The lighter lanthanide ions (till Ho^3+^) favored extended lanthanide cluster coordination interaction forming 1D chains, while the heavier ones (Er and above) resulted in a stacking of a cation, Anderson-Evans cluster derivatised by a pair of lanthanide hydrates and an anion, a discrete Anderson-Evans clusters through H-bonding (0D). In both series, lattice water provides further stability to the structure. Observation of purple color in all chromium molybdate cluster based solids reported here as well as in literature is attributed to the domination of Cr^3+^ emission. Quenching of lanthanide emission in rare-earth coordinated chromium based Anderson-Evans cluster can be ascribed to energy transfer from 4f levels of Ln^3+^ ions to that of Cr^3+^*d*-orbital energy levels. Further theoretical calculations are required to quantify the same. A future problem is to extend our methodology to assemble aluminum based cluster anion with two or more emitting lanthanide ions, i.e., possible solid solutions to realize white light materials. The paper highlights that structure-property evaluation of a POM system is more meaningful toward the design of functional materials if one adopts a crystal engineering approach to rationalize the fundamental chemistry.

## Author Contributions

AR conceived the idea as he has been working in this field for over 20 years. ST has the lead role in designing the synthesis and characterization. B and VK helped in the refinement crystal structures and supported in data collection and analysis. GJ supported in synthesis and data collection. MA carried out the photoluminescence experiments and related tasks. GVP carried out the photoluminescence studies and related tasks.

### Conflict of Interest Statement

The authors declare that the research was conducted in the absence of any commercial or financial relationships that could be construed as a potential conflict of interest.

## Supplementary Material

In Supplementary Material, Table 1 contains crystallographic information files (CIF) for all unreported solids, their Rietveld refinement plots (Figure S3), IR plots for all the synthesized solids (Figures S4, S5), Thermal Analysis plots for selected solids (Figure S6), Emission spectra of TbAl23 from commercial spectrofluorophotometer (Figure S7), the emission spectra for TmCr13 and TmAl27 is given in Figure S8. Figure S9 shows the emission comparison of EuCr7 and NdCr5. The emission spectra of other Cr-based solids and their corresponding Bright Field and Photoluminescence images are shown in Figure S10. Rietveld refined parameters of all unreported solids (Table S1). Table S2 has crystal structure and refinement parameters of reported solids that were resynthesized. IR frequencies (Table S3). Bond Valance Sum calculations for TbAl23 and TmAl27 (Table S4). Table S5 contains the yields for all solids synthesized and Table S6 reports selected bond distances and angles for all unreported solids. Optical data for all solids (Figures 4, S10). **Image 1** contains the Graphical Abstract which shows pictures of all the solids prepared for this study taken from Nikon D3300 DSLR camera (middle) and Nikon SMZ-745T microscope (top and bottom). A clear pink color is visible to eye can be seen in chromium analogs whereas in aluminum analogs, the typical color of corresponding lanthanides can be seen.
