# Supplementary material for: Photoluminescence Properties of Two Closely Related Isostructural Series Based on Anderson-Evans Cluster Coordinated With Lanthanides [Ln(H2O)7{X(OH)6Mo6O18}]•yH2O, X = Al, Cr
Source: Front Chem. 2019 Jan 7;6:631. doi: 10.3389/fchem.2018.00631 (PMC6330572; doi:10.3389/fchem.2018.00631)
Supplement: Supplementary file 1 [file Table_1.docx]

Supplementary Material

Photoluminescence properties of two closely related isostructural series based on Anderson-Evans cluster coordinated with lanthanides [Ln(OH_2_)_7_{X(OH)_6_Mo_6_O_18_}].yH_2_O, X=Al, Cr

Shailabh Tewari^1^, Mohammad Adnan^2^, Balendra^1^, Vineet Kumar^1^, Gaurav Jangra^1^, G Vijaya Prakash^2*^, Arunachalam Ramanan^1*^

^1^Solid state and Materials chemistry Laboratory, Department of Chemistry, Indian Institute of Technology Delhi, HauzKhas, New Delhi, India

^2^Nanohotonics Laboratory, Department of Physics, Indian Institute of Technology Delhi, HauzKhas, New Delhi, India

*** Correspondence:**Arunachalam Ramanan
aramanan@chemistry.iitd.ac.in

G. Vijaya Prakash

prakash@physics.iitd.ac.in

**Fig. S1.** (a) The building block in Series I, [Tb(OH_2_)_7_{Al(OH)_6_Mo_6_O_18_}].4H_2_O (b) The nine-coordinated Tb coordinates with the cluster oxygens O24 and O20 extending into 1D chains. Coordination of lanthanide with terminal oxygens of the cluster at 1,3 position w.r.t. Mo atoms forming the hexagonal ring. Adjacent chains interact through O_Mo_−H∙∙∙Ow. Lattice water molecules also facilitate H-bonding interactions.

**Fig. S2.** (a)The complex cation, [{(H_2_O)_7_Tm{Al(OH)_6_Mo_6_O_18_}Tm(OH_2_)_7_]^3+^ and the cluster anion {Al(OH)_6_Mo_6_O_18_}^3-^are the building blocks in Series II along with 16 lattice water molecules. (b) The ions aggregate through H-bonding interactions. Series II is an example of a molecular solid (0D). The anionic Anderson-Evans cluster is depicted in orange for clarity.

**Fig. S3.** Rietveld refinement plot for all the new solids reported in the work. The blue corresponds to the experimental data while the red represents calculated profile.

**Fig. S4(a-x).** FT-IR spectra of all the solids recorded with Nicolet 5DX spectrometer

**Fig. S5.** FT-IR for solids recorded with Nicolet 6700 spectrometer

**Fig. S6.** Thermal Analysis plots for (a)DyCr10, (b)HoCr11,(c)ErCr12, (d)TmCr13, (e)YbCr14, (f)PrAl18, (g)NdAl19, (h)TbAl23, (i)DyAl24, (j)ErAl26, (k) TmAl27 and (l) YbAl28.

In most of the samples, artifacts like mass gain are observed above 500 ^o^C. Several factors like buoyancy effects, chemical reactions leading to the formation of hardly volatile compounds and adsorption of gaseous substances onto the sample could have caused a fluctuation in the mass.

**Fig. S7.** The emission spectra of TbAl23 at various excitations . Excitation spectra monitored at 543nm is also given at the top ( gray color). The emisssion spectra when excited at 400 nm gives no emission characteristics as evident from the excitatin spectra that there is no absrption energy level at this excitation. The excitation source is a Xe lamp coupled to a monochromator ( see text).

**Fig. S8.** The emission spectra of TmCr13 and TmAl27 when excited at 400 nm diode laser. TmAl27 shows very low emission in UV-violet region, along with the substrate emission ( marked in the gray shaded area). The corresponding bright field (BF) and PL images are also shown.

**Fig. S9.** Emission spectra of EuCr7 and NdCr5 and their corresponding PL images. The spectra are recorded under similar experimental condition (excited by 400nm diode laser, ~ 10mW) and the intensity of NdCr5 is about 4.6% with respect to EuCr7.

**Fig. S10.** The emission epectra of infrared rare-earth containing Chromium molybdate complexes. The corresponding bright field (BF) and Photoluminescence (PL) images are shown (leftside of each emission spectra). Excited by 400nm diode laser ( see text).

| **Solid** | **Refined cell parameters ( from reitveld analysis)** | **Refined cell parameters ( from single-crystal data)** |
| --- | --- | --- |
| **TbCr9** | *P*ca2_1_  a (Å) = 11.78032(73)  b (Å) = 10.91660(83)  c (Å) = 22.30377(91)  V (Å^3^) = 2868.29(31)  Rp = 7.11  Rwp = 10.46 | *P*ca2_1_  a (Å) = 11.8213(7)  b (Å) =10.9479(6)  c (Å) = 22.3810(13)  V (Å^3^) = 2896.5(3) |
| **DyCr10** | *P*ca2_1_  a (Å) = 11.7813(2)  b (Å) = 10.9100(5)  c (Å) = 22.282(11)  V (Å^3^) = 2864.0(7)  Rp = 4.39  Rwp = 5.91 | *P*ca2_1_  a (Å) = 11.7813(7)  b (Å) =10.9100(6)  c (Å) = 22.2821(13)  V (Å^3^) = 2864.0(3) |
| **HoCr11** | *P*ca2_1_  a (Å) = 11.769(61)  b (Å) = 10.882(19)  c (Å) = 22.228(32)  V (Å^3^) = 2847.16(9)  Rp = 5.51  Rwp = 8.28 | *P*ca2_1_  a (Å) = 11.7696(10)  b (Å) =10.88299(9)  c (Å) = 22.2283(18)  V (Å^3^) = 2847.2(4) |
| **ErCr12** | *P* ī  a (Å) = 11.177(5)  b (Å) = 11.650(10)  c (Å) = 13.8960(3)  α (°) = 74.439(5)  β (°) = 83.805(5)  γ (°) = 89.457(5)  V (Å^3^) = 1732.68(11)  Rp = 8.27  Rwp = 12.05 | *P* ī  a (Å) = 10.9762(6)  b (Å) =11.6149(7)  c (Å) = 13.9397(8)  α (°) = 74.4390(10)  β (°) = 83.8050(10)  γ (°) = 89.4570(10)  V (Å^3^) = 1701.65(17) |
| **PrAl18** | *P*ca2_1_  a (Å) = 11.8997(3)  b (Å) = 10.9215(7)  c (Å) = 22.7267(2)  V (Å^3^) = 2953.6(1)  Rp = 6.85  Rwp = 9.69 | *P*ca2_1_  a (Å) = 11.8173(6)  b (Å) =10.9707(5)  c (Å) = 22.5088(11)  V (Å^3^) = 2918.1(2) |
| **NdAl19** | *P*ca2_1_  a(Å) = 11.759(5)  b (Å) = 10.9253(3)  c (Å) = 22.3965(4)  V (Å^3^) = 2877.4(10)  Rp = 7.08  Rwp = 10.60 | *P*ca2_1_  a (Å) = 11.7659(5)  b (Å) =10.9321(5)  c (Å) = 22.4151(10)  V (Å^3^) = 2883.2(2) |
| **TbAl23** | *P*ca2_1_  a (Å) = 11.771(21)  b (Å) = 10.945(24)  c (Å) = 22.263(21)  V (Å^3^) = 2868.3(8)  Rp = 6.34  Rwp = 9.15 | *P*ca2_1_  a (Å) = 11.7860(13)  b (Å) =10.9560(11)  c (Å) = 22.333(2)  V (Å^3^) = 2883.9(5) |
| **DyAl24** | *P*ca2_1_  a (Å) = 11.781(3)  b (Å) = 10.910(10)  c (Å) = 22.282(10)  V (Å^3^) = 2864.0(3)  Rp = 5.60  Rwp = 7.86 | *P*ca2_1_  a (Å) = 11.7385(5)  b (Å) =10.8989(5)  c (Å) = 22.2203(10)  V (Å^3^) = 2842.8(2) |
| **HoAl25** | *P*ca2_1_  a (Å) = 11.777(4)  b (Å) = 10.926(15)  c (Å) = 22.218(9)  V (Å^3^) = 2859.1(7)  Rp = 6.18  Rwp = 8.25 | *P*ca2_1_  a (Å) = 11.7114(11)  b (Å) =10.8538(10)  c (Å) = 22.139(2)  V (Å^3^) = 2814.2(5) |
| **ErAl26#** | *P*ca2_1_  a (Å) = 11.7567(13)  b (Å) = 10.9011(23)  c (Å) = 22.1977(14)  V (Å^3^) = 2844.8(9)  Rp = 8.8  Rwp = 16.66 | *P*ca2_1_  a (Å) = 11.667(18)  b (Å) =10.846(18)  c (Å) = 22.11(4)  V (Å^3^) = 2797(8) |
| **TmAl27** | *P* ī  a (Å) = 11.2084(8)  b (Å) = 11.6212(3)  c (Å) = 13.9788(11)  α (°) = 74.43(10)  β (°) = 83.82 (10)  γ (°) = 89.449(5)  V (Å ^3^) = 1743.4(27)  Rp = 8.99  Rwp = 13.41 | *P* ī  a (Å) = 10.9836(4)  b (Å) =11.5857(4)  c (Å) = 13.9171(5)  α (°) = 74.4310(10)  β (°) = 83.8200(10)  γ (°) = 89.4490(10)  V (Å ^3^) = 1695.75(11) |

**Table S1.** Rietveld refined perameters of all unreported solids.

| Parameter | **NdCr5** | **EuCr7** | **YbCr14** | **CeAl17** | **YbAl28** |
| --- | --- | --- | --- | --- | --- |
| Formula | Cr_2_Mo_12_Nd_2_O_62_, 8(O) | Cr_2_Eu_2_Mo_12_O_62_,  8(O) | CrMo_6_O_38_Yb_2_,Cr Mo_6_O_24_,16(O) | Al_2_Ce_2_Mo_12_ O_62_,8(O) | AlMo_6_O_38_Yb_2_,Al Mo_6_O_24_16(O)' |
| Formula weight | 2791.10 | 2679.22 | 2849.36 | 2605.48 | 2799.32 |
| T (K) | 100 | 298 | 298 | 298 | 298 |
| Crystal system | Orthorhombic | Orthorhombic | Triclinic | Orthorhombic | Triclinic |
| Space group | *P*ca2_1_ | *P*ca2_1_ | *P* ī | *P*ca2_1_ | *P* ī |
| *a* (Ǻ) | 11.782(2) | 10.961(10) | 11.0379(5) | 11.8255(15) | 11.011(10) |
| *b* (Ǻ) | 10.9543(18) | 11.778(10) | 11.6436(5) | 11.0007(14) | 11.585(12) |
| *c* (Ǻ) | 22.407(6) | 22.422(19) | 13.9044(7) | 22.556(3) | 13.885(13) |
| *α* (°) | 90 | 90 | 75.0050(10) | 90 | 74.895(19) |
| *β* (°) | 90 | 90 | 84.5300(10) | 90 | 84.51(2) |
| *γ* (°) | 90 | 90 | 89.4660(10) | 90 | 89.517(18) |
| V (Å^3^) | 2892.0(10) | 2895(4) | 1718.08(14) | 2934.3(6) | 1702(3) |
| Z | 2 | 2 | 1 | 2 | 1 |
| Dcalc (g.cm^-3^) | 3.059 | 3.074 | 2.754 | 2.949 | 2.731 |
| *μ*_MoKα_ (cm^-1^) | 5.871 | 5.128 | 5.232 | 4.153 | 5.005 |
| Theta range (°) | 2.50, 31.39 | 2.54, 25.68 | 2.51, 30.88 | 2.53, 24.03 | 2.29, 26.17 |
| R_1_, wR_2_ [I > 2σ(I)]^a^ | 0.0477, 0.1379 | 0.0280, 0.0892 | 0.0371, 0.1345 | 0.0361, 0.1124 | 0.0360, 0.1243 |
| GOF | 1.101 | 0.724 | 1.001 | 0.947 | 0.938 |

**Table S2.** Crystal structure and refinement parameters of reported solids that were resynthesized.

| **Solids** | **IR frequencies (cm-1 )** | | | | |
| --- | --- | --- | --- | --- | --- |
|  | **Mo-O_t_** | **Mo-O_b_-Mo** | **Mo-O_c_-X** | **coordinated and lattice water** | **deformation vibrations of HOH** |
| **Chromium based solids** | | | | | |
| **YCr1** | 943.04  900.6 | 640.26 | 426.19 | 3459.724 | 1612.224 |
| **LaCr2** | 944.539  894.540 | 654.082 | 416.731 | 3368.786 | 1626.213 |
| **CeCr3** | 946.502  898.187 | 659.819 | 415.381 | 3446.700 | 1621.473 |
| **PrCr4** | 946.644  899.411 | 659.640 | 417.167 | 3375.945 | 1621.816 |
| **NdCr5** | 946.921  899.390 | 659.158 | 416.867 | 3440.788 | 1620.808 |
| **SmCr6** | 946.821  899.075 | 657.992 | 416.119 | 3455.907 | 1618.449 |
| **EuCr7** | 946.297  899.371 | 657.980 | 416.960 | 3370.181 | 1618.574 |
| **GdCr8** | 946.909  899.159 | 657.572 | 416.572 | 3460.947 | 1617.927 |
| **TbCr9** | 947.077  899.070 | 656.687 | 416.757 | 3459.661 | 1617.539 |
| **DyCr10** | 947.307  897.471 | 653.071 | 415.335 | 3367.110 | 1617.077 |
| **HoCr11** | 946.556  896.707 | 652.981 | 416.662 | 3363.480 | 1617.362 |
| **ErCr12** | 946.417  896.522 | 650.723 | 417.299 | 3362.826 | 1620.373 |
| **TmCr13** | 946.89  896.75 | 665.33 | 424.27 | 3396.083 | 1616.081 |
| **YbCr14** | 947.310  896.585 | 649.953 | 417.841 | 3335.395 | 1621.436 |
| **Aluminium based solids** | | | | | |
| **YAl15** | 948.82  900.61 | 657.61 | 437.76 | 3382.584 | 1614.152 |
| **LaAl16** | 948.109  903.671 | 659.004 | 447.456 | 3341.218 | 1626.364 |
| **CeAl17** | 946.721  905.831 | 662.306 | 449.068 | 3339.737 | 1625.232 |
| **PrAl18** | 946.963  905.931 | 663.312 | 448.299 | 3349.243 | 1624.847 |
| **NdAl19** | 941.573  900.433 | 665.144 | 446.463 | 3357.167 | 1619.376 |
| **SmAl20** | 946.867  901.390 | 665.639 | 447.120 | 3349.802 | 1617.853 |
| **EuAl21** | 940.841  901.673 | 665.902 | 447.880 | 3368.895 | 1619.170 |
| **GdAl22** | 940.973  901.082 | 664.259 | 446.890 | 3359.558 | 1617.391 |
| **TbAl23** | 946.993  898.460 | 665.338 | 445.900 | 3486.631 | 1619.496 |
| **DyAl24** | 946.867  901.077 | 664.095 | 447.713 | 3355.838 | 1617.352 |
| **HoAl25** | 940.499  900.993 | 661.205 | 447.724 | 3328.587 | 1617.286 |
| **ErAl26** | 895.943 | 654.906 | 446.335 | 3366.278 | 1623.074 |
| **TmAl27** | 954.61  910.25 | 682.68 | 433.91 | 3369.084 | 1612.224 |
| **YbAl28** | 924.524  896.653 | 653.676 | 448.961 | 3343.911 | 1627.550 |

**Table S3.** IR frequencies.

| **TbAl23** | | |
| --- | --- | --- |
| **Metal** | **Bond Valence Calculations** | **BV-sum; BV sum deviation** |
| **Tb1** | Tb1-O26: 2.3856Å s=0.3714v.u.  Tb1-O24: 2.4681Å s=0.3070v.u.  Tb1-O29: 2.5138Å s=0.2762v.u.  Tb1-O27: 2.3974Å s=0.3615v.u.  Tb1-O20: 2.5701Å s=0.2426v.u.  Tb1-O30: 2.4272Å s=0.3374v.u.  Tb1-O25: 2.3740Å s=0.3815v.u.  Tb1-O28: 2.3849Å s=0.3720v.u.  Tb1-O31: 2.4284Å s=0.3365v.u. | 3.0268v.u. ; 0.0268v.u. |
| **Al1** | Al1-O2: 1.9041Å s=0.4870v.u.  Al1-O5: 1.8835Å s=0.5112v.u.  Al1-O3: 1.9074Å s=0.4832v.u.  Al1-O1: 1.9092Å s=0.4811v.u.  Al1-O6: 1.9172Å s=0.4722v.u.  Al1-O4: 1.8943Å s=0.4984v.u. | 3.0504v.u. ; 0.0504v.u. |
| **Mo1** | Mo1-O2: 2.2965Å s=0.3715v.u.  Mo1-O7: 1.9537Å s=0.8928v.u.  Mo1-O1: 2.2699Å s=0.3977v.u.  Mo1-O14: 1.7135Å s=1.6501v.u.  Mo1-O8: 1.8833Å s=1.0689v.u.  Mo1-O13: 1.7125Å s=1.6542v.u. | 6.1085v.u. ; 0.1085v.u. |
| **Mo2** | Mo2-O2: 2.2987Å s=0.3694v.u.  Mo2-O3: 2.3119Å s=0.3571v.u.  Mo2-O15: 1.7132Å s=1.6514v.u.  Mo2-O8: 1.9689Å s=0.8586v.u.  Mo2-O16: 1.6935Å s=1.7369v.u.  Mo2-O9: 1.9286Å s=0.9520v.u. | 5.9991v.u. ; -0.0009v.u. |
| **MO3** | Mo3-O3: 2.3124Å s=0.3567v.u.  Mo3-O10: 1.9688Å s=0.8590v.u.  Mo3-O4: 2.2782Å s=0.3893v.u.  Mo3-O18: 1.6917Å s=1.7448v.u.  Mo3-O17: 1.7056Å s=1.6838v.u.  Mo3-O9: 1.9279Å s=0.9536v.u. | 6.0577v.u. ; 0.0577v.u. |
| **Mo4** | Mo4-O11: 1.9290Å s=0.9510v.u.  Mo4-O5: 2.3191Å s=0.3507v.u.  Mo4-O10: 1.9462Å s=0.9099v.u.  Mo4-O4: 2.2887Å s=0.3790v.u.  Mo4-O19: 1.7127Å s=1.6535v.u.  Mo4-O20: 1.7198Å s=1.6236v.u. | 5.9664v.u. ; -0.0336v.u. |
| **Mo5** | Mo5-O12: 1.9482Å s=0.9054v.u.  Mo5-O11: 1.9033Å s=1.0157v.u.  Mo5-O5: 2.3160Å s=0.3534v.u.  Mo5-O21: 1.7092Å s=1.6682v.u.  Mo5-O6: 2.2779Å s=0.3896v.u.  Mo5-O22: 1.6939Å s=1.7352v.u. | 6.1496v.u. ; 0.1496v.u. |
| **Mo6** | Mo1-O2: 2.2965Å s=0.3715v.u.  Mo1-O7: 1.9537Å s=0.8928v.u.  Mo1-O1: 2.2699Å s=0.3977v.u.  Mo1-O14: 1.7135Å s=1.6501v.u.  Mo1-O8: 1.8833Å s=1.0689v.u.  Mo1-O13: 1.7125Å s=1.6542v.u. | 5.9969v.u. ; -0.0031v.u. |
| **TmAl27** | | |
| **Metal** | **Bond Valence Calculations** | **BV-sum; BV sum deviation** |
| **Tm1** | Tm1-O2: 2.3813Å s=0.3581v.u.  Tm1-O1: 2.3787Å s=0.3604v.u.  Tm1-O5: 2.3913Å s=0.3497v.u.  Tm1-O3: 2.2944Å s=0.4403v.u.  Tm1-O7: 2.2813Å s=0.4542v.u.  Tm1-O4: 2.3292Å s=0.4054v.u.  Tm1-O8: 2.2939Å s=0.4408v.u.  Tm1-O6: 2.3254Å s=0.4090v.u. | 3.2607v.u. ; 0.2607v.u. |
| **Al1** | Al1-O59: 1.8871Å s=0.5069v.u.; 1.8871Å s=0.5069v.u.  Al1-O61: 1.9110Å s=0.4791v.u.; 1.9111Å s=0.4790v.u.  Al1-O57: 1.9049Å s=0.4861v.u. ; 1.9049Å s=0.4861v.u. | 3.0638v.u. ; 0.0638v.u. |
| **Al2** | Al2-O37: 1.8951Å s=0.4974v.u.; 1.8951Å s=0.4974v.u.  Al2-O35: 1.9071Å s=0.4835v.u.; 1.9071Å s=0.4836v.u.  Al2-O36: 1.8958Å s=0.4966v.u. ; 1.8958Å s=0.4965v.u. | 3.0743v.u. ; 0.0743v.u. |
| **Mo1** | Mo1-O61: 2.2725Å s=0.3951v.u.  Mo1-O57: 2.3196Å s=0.3502v.u.  Mo1-O53: 1.9237Å s=0.9639v.u.  Mo1-O56: 1.9168Å s=0.9810v.u.  Mo1-O55: 1.7120Å s=1.6567v.u.  Mo1-O54: 1.6993Å s=1.7112v.u. | 6.1262v.u. ; 0.1262v.u. |
| **Mo2** | Mo2-O59: 2.2686Å s=0.3990v.u.  Mo2-O61: 2.2860Å s=0.3816v.u.  Mo2-O41: 1.9404Å s=0.9236v.u.  Mo2-O53: 1.9283Å s=0.9528v.u.  Mo2-O51: 1.6962Å s=1.7248v.u.  Mo2-O52: 1.7113Å s=1.6593v.u. | 6.1248v.u. ; 0.1248v.u. |
| **Mo6** | Mo6-O59: 2.2830Å s=0.3845v.u.  Mo6-O57: 2.3099Å s=0.3590v.u.  Mo6-O41: 1.9325Å s=0.9424v.u.  Mo6-O56: 1.9425Å s=0.9186v.u.  Mo6-O40: 1.7019Å s=1.6998v.u.  Mo6-O39: 1.6873Å s=1.7647v.u. | 6.1450v.u. ; 0.1450v.u. |
| **Mo9** | Mo9-O37: 2.2773Å s=0.3902v.u.  Mo9-O13: 1.9349Å s=0.9366v.u.  Mo9-O35: 2.2996Å s=0.3686v.u.  Mo9-O10: 1.9366Å s=0.9326v.u.  Mo9-O11: 1.7042Å s=1.6899v.u.  Mo9-O12: 1.7037Å s=1.6922v.u. | 6.0862v.u. ; 0.0862v.u. |
| **Mo10** | Mo10-O35: 2.2908Å s=0.3770v.u.  Mo10-O10: 1.9216Å s=0.9690v.u.  Mo10-O36: 2.3168Å s=0.3527v.u.  Mo10-O24: 1.9204Å s=0.9722v.u.  Mo10-O2: 1.7246Å s=1.6040v.u.  Mo10-O9: 1.6867Å s=1.7673v.u. | Mo10:6.1195v.u. ; 0.1195v.u. |
| **Mo12** | Mo12-O37: 2.2687Å s=0.3989v.u.  Mo12-O13: 1.9398Å s=0.9252v.u.  Mo12-O36: 2.2856Å s=0.3820v.u.  Mo12-O24: 1.9327Å s=0.9420v.u.  Mo12-O23: 1.7054Å s=1.6845v.u.  Mo12-O22: 1.6977Å s=1.7183v.u. | 6.1293v.u. ; 0.1293v.u. |

**Table S4.** Bond valence sum calculations for TbAl23 and TmAl27.

| **Solid** | **Cr** | | | **Solid** | **Al** | | |
| --- | --- | --- | --- | --- | --- | --- | --- |
|  | **Yield**  **(gm)** | **Theoretical**  **Yield**  **(gm)**  **(based on Mo)** | **%yield**  **(based on Mo)** |  | **Yield**  **(gm)** | **Theoretical**  **Yield**  **(gm)**  **(based on Mo)** | **%yield**  **(based on Mo)** |
| **LaCr2** | 0.1371 | 0.1468 | 93 | LaAl16 | 0.1168 | 0.1440 | 81 |
| **CeCr3** | 0.1363 | 0.1469 | 92 | CeAl17 | 0.1270 | 0.1442 | 88 |
| **PrCr4** | 0.1155 | 0.1470 | 78 | PrAl18 | 0.1124 | 0.1443 | 77 |
| **NdCr5** | 0.1248 | 0.1473 | 84 | NdAl19 | 0.1220 | 0.1446 | 8 |
| **SmCr6** | 0.1297 | 0.1480 | 87 | SmAl20 | 0.1214 | 0.1453 | 83 |
| **EuCr7** | 0.1364 | 0.1482 | 92 | EuAl21 | 0.1140 | 0.1455 | 78 |
| **GdCr8** | 0.1303 | 0.1487 | 87 | GdAl22 | 0.1159 | 0.1460 | 79 |
| **TbCr9** | 0.1166 | 0.1489 | 78 | TbCr23 | 0.1304 | 0.1462 | 89 |
| **DyCr10** | 0.1074 | 0.1493 | 71 | DyCr24 | 0.1115 | 0.1466 | 76 |
| **HoCr11** | 0.1108 | 0.1496 | 74 | HoCr25 | 0.1245 | 0.1469 | 84 |
| **ErCr12** | 0.1300 | 0.1576 | 82 | ErCr26 | 0.1218 | 0.1549 | 78.6 |
| **YbCr14** | 0.1284 | 0.1583 | 81 | YbCr28 | 0.1188 | 0.1555 | 76.4 |

**Table S5.** Yields for reported solids.

| **TbCr9** | | | |
| --- | --- | --- | --- |
| **Tb-O** | **2.346-2.556** | **O-Mo-O(Type-I)** | **69.71-106.81** |
| **Mo-O** | **1.689-1.734** | **O-Mo-O(Type II)** | **147.81-164.06** |
| **Mo-O (Type -II)** | **1.898-1.974** | **O-Cr-O (Type I)** | **83.73-96.34** |
| **Mo-O (Type -III)** | **2.262-2.213** | **O-Cr-O(Type-II)** | **179.67-179.68** |
| **Cr-O** | **1.954-1.974** | **Mo-O-Mo** | **91.66-120.48** |
| **O-Tb-O (Type-I)** | **113.88-139.94** | **Cr-O-Mo** | **101.45-103.79** |
| **O-Tb-O (Type-II)** | **66.02-93.05** | **M-O-Tb** | **152.79-165.4** |
| **DyCr10** | | | |
| **Dy-O** | **2.332-2.547** | **O-Mo-O(Type-I)** | **70.29-105.7** |
| **Mo-O** | **1.696-1.728** | **O-Mo-O(Type-II)** | **149.45-162.44** |
| **Mo-O (Type-II)** | **1.884-1.96** | **O-Cr-O(Type-I)** | **83.34-119.87** |
| **Mo-O (Type-III)** | **2.252-2.313** | **O-Cr-O(Type-II)** | **179.03-179.78** |
| **Cr-O** | **1.958-1.98** | **Mo-O-Mo** | **91.66-119.87** |
| **O-Dy-O(Type-I)** | **70.83-93.42** | **Cr-O-Cr** | **101.68-103.42** |
| **O-DY-O(Type-II)** | **114.18-140.31** | **Mo-O-Dy** | **152.42-166.25** |
| **HoCr11** | | | |
| **Ho-O** | **2.283-2.540** | **O-Mo-O(Type-I)** | **69.78-108.18** |
| **Mo-O** | **1.683-1.718** | **O-Mo-O(Type-II)** | **147.9-162.78** |
| **Mo-O (Type-II)** | **1.887-1.962** | **O-Cr-O(Type-I)** | **83.16-96.66** |
| **Mo-O (Type-III)** | **2.225-2.314** | **O-Cr-O(Type-II)** | **179.28-179.78** |
| **Cr-O** | **1.929-1.991** | **Cr-O-Mo** | **101.24-104.34** |
| **O-Ho-O(Type-I)** | **66.09-94.02** | **Mo-O_Mo** | **91.79-119.78** |
| **O-Ho-O(Type-II)** | **111.92-139.68** | **Mo-O-Ho** | **152.79-166.87** |
| **ErCr12** | | | |
| **Er-O** | **2.302-2.542** | **O-Er-O(Type-II)** | **140.32-148.67** |
| **Mo-O** | **1.691-1.723** | **O-Mo-O (Type-I)** | **70.98-106.41** |
| **Mo-O (Type-II)** | **1.910-1.946** | **O-Mo-O (Type-II)** | **147.99-161.75** |
| **Mo-O (Type-III)** | **2.241-2.306** | **O-Cr-O** | **83.36-96.53** |
| **Cr-O** | **1.960-1983** | **Cr-O-Mo** | **101.31-103.13** |
| **O-O (Type-I)** | **.612-.849** | **Mo-O-Mo** | **92.46-119.37** |
| **O-O (Type-II)** | **1.258-1.674** | **O-O-Er** | **60.36-106.28** |
| **O-Er-O (Type-I)** | **69.39-128.18** | **Mo-O-Er** | **160.14** |
| **TmCr13** | | | |
| **Tm-O** | **2.232-2.505** | **O-Mo-O (Type-I)** | **68.62-107.98** |
| **Mo-O** | **1.667-1.74** | **O-Mo-O (Type-II)** | **147.54-163.18** |
| **Mo-O (Type-II)** | **1.871-1.985** | **O-Cr-O (Type-I)** | **82.78-96.56** |
| **Mo-O (Type-II)** | **2.259-2.320** | **O-Cr-O (Type-II)** | **178.35-178.54** |
| **Cr-O** | **1.943-1.972** | **Cr-O-Mo** | **101.81-105.12** |
| **O-Tm-O (Type-I)** | **70.96-111.69** | **Mo-O-Mo** | **91.36-120.56** |
| **O-Tm-O (Type-II)** | **122.74-141.12** | **Mo-O-Tm** | **154.04-164.07** |
| **Tm-O** | **2.232-2.505** | **O-Mo-O (Type-I)** | **68.62-107.98** |
| **PrAl18** | | | |
| **Pr-O** | **2.435-2.630** | **O-Mo-O (Type –I)** | **66.74-107.32** |
| **Mo-O** | **1.689-1.728** | **O-Mo-O (Type –II)** | **149.25-162.51** |
| **Mo-O (Type-II)** | **1.896-1.969** | **O-Al-O (Type –I)** | **84.16-96.15** |
| **Mo-O (Type-III)** | **2.269-2.328** | **O-Al-O (Type-II)** | **178.81-179.54** |
| **Al-O** | **1.894-1.902** | **Mo-O-Mo** | **90.73-119.95** |
| **O-Pr-O (Type-I)** | **65.34-114.32** | **Al-O-Mo** | **102.78-104.76** |
| **O-Pr-O (Type-II)** | **133.43-141.22** | **Mo-O-Pr** | **151.1-165.51** |
| **NdAl19** | | | |
| **Nd-O** | **2.397-2.62** | **O-Mo-O (Type-I)** | **67.75-107.16** |
| **Mo-O** | **1.69-1.720** | **O-Mo-O (Type-II)** | **149.03-162.89** |
| **Mo-O (Type-II)** | **1.886-1.974** | **O-Al-O (Type-I)** | **84.23-96.36** |
| **Mo-O (Type-III)** | **2.270-2.325** | **O-Al-O (Type-II)** | **178.8-178.91** |
| **Al-O** | **1.879-1.917** | **Mo-O-Mo** | **90.35-119.34** |
| **O-Nd-O (Type-I)** | **65.49-95.96** | **Al-O-Mo** | **102.83-104.72** |
| **O-Nd-O (Type-II)** | **118.63-141.61** | **Mo-O-Nd** | **150.96-166.01** |
| **DyAl24** | | | |
| **Dy-O** | **2.335-2.563** | **O-Mo-O (Type-I)** | **67.10-107.46** |
| **Mo-O** | **1.699-1.720** | **O-Mo-O (Type-II)** | **149.29-162.95** |
| **Mo-O (Type-II)** | **1.883-1.969** | **O-Al-O (Type-I)** | **83.96-96.28** |
| **Mo-O (Type-III)** | **2.257-2.316** | **O-Al-O (Type-II)** | **178.41-178.94** |
| **Mo-OH** | **1.901-1.920** | **Mo-O-Mo** | **90.6-119.09** |
| **Al-O** | **1.873-1.914** | **Al-O-Mo** | **102.84-104.54** |
| **O-Dy-O (Type-I)** | **67.31-93.2** | **Mo-O-Py** | **152.28-165.18** |
| **O-Dy-O (Type-II)** | **113.11-140.69** | **O-Mo-O (Type-I)** | **67.10-107.46** |
| **HoAl25** | | | |
| **Ho-O** | **1.948-2.633** | **O-Mo-O (Type-I)** | **66.60-109.93** |
| **Mo-O** | **1.655-1.758** | **O-Mo-O (Type-II)** | **147.46-163.77** |
| **Mo-O (Type-II)** | **1.871-1.979** | **O-Al-O (Type-I)** | **83.68-97.29** |
| **Mo-O (Type-III)** | **2.252-2.344** | **O-Al-O (Type-II)** | **177.12-177.48** |
| **Al-O** | **1.845-1.945** | **Al-O-Mo** | **101.04-105.95** |
| **O-O** | **.772** | **Mo-O-Mo** | **90.32-118.72** |
| **O-Ho-O (Type-I)** | **62.19-95.04** | **Mo-O-Ho** | **150.47-165.42** |
| **ErAl26** | | | |
| **Er-O** | **2.289-2.542** | **O-Mo-O (Type-I)** | **67.51-107.09** |
| **Mo-O** | **1.634-1.722** | **O-Mo-O (Type-II)** | **148.38-164.02** |
| **Mo-O (Type-II)** | **1.887-1.977** | **O-Al-O (Type-I)** | **83.65-96.16** |
| **Mo-O (Type-III)** | **2.241-2.334** | **O-Al-O (Type-II)** | **177.08-178.06** |
| **Al-O** | **1.881-1.907** | **Mo-O-Mo** | **89.78-119.17** |
| **O-Er-O (Type-I)** | **64.05-94.43** | **Al-O-Mo** | **101.46-104.9** |
| **O-Er-O (Type-II)** | **112.69-141.1** | **Mo-O-Er** | **157.33-164.17** |
| **ErAl26** | | | |
| **Er-O** | **2.284-2.474** | **O-Mo-O (Type-I)** | **67.51-107.09** |
| **Mo-O** | **1.7-1.724** | **O-Mo-O (Type-II)** | **147.98-164.02** |
| **Mo-O (Type-II)** | **1.912-1.958** | **O-Al-O (Type-I)** | **84.01-96.16** |
| **Mo-O (Type-III)** | **2.260-2.314** | **O-Al-O (Type-II)** | **177.08-179.76** |
| **Al-O** | **1.875-1.915** | **Mo-O-Mo** | **89.78-119.17** |
| **O-O** | **1.697** | **Al-O-Mo** | **101.46-104.9** |
| **O-Er-O (Type-I)** | **69.8-123.79** | **Mo-O-Er** | **157.33-164.17** |
| **O-Er-O (Type-II)** | **133.72-149.88** |  |  |
| **ErAl26*** | | | |
| **Er-O** | **2.297-2.429** | **O-Mo-O (Type-I)** | **67.68-106.72** |
| **Mo-O** | **1.698-1.724** | **O-Mo-O (Type-II)** | **147.98-161.05** |
| **Mo-O (Type-II)** | **1.912-1.958** | **O-Al-O (Type-I)** | **84.22-96.24** |
| **Mo-O (Type-III)** | **2.260-2.336** | **O-Al-O (Type-II)** | **179.16-179.52** |
| **Al-O** | **1.875-1.915** | **Mo-O-Mo** | **91.24-118.23** |
| **O-Er-O (Type-I)** | **41.55-47.6** | **Al-O-Mo** | **102.04-104.97** |
| **O-Er-O (Type-II)** | **69.8-125.79** | **O-O-Er** | **63.24-75.22** |
| **O-Er-O (Type-III)** | **138.24-149.88** | **Mo-O-Er** | **158.19-161.2** |
| **Tm-Al27** | | | |
| **Tm-O** | **2.280-2.681** | **O-Tm-O (Type-II)** | **137.25-148.41** |
| **Mo-O** | **1.696-1.727** | **O-Mo-O (Type-i)** | **72.55-106.58** |
| **Mo-O (Type-II)** | **1.903-1.953** | **O-Mo-O (Type-II)** | **148.84-160.53** |
| **Mo-O (Type-III)** | **2.276-2.306** | **O-Al-O** | **84.44-95.66** |
| **Al-O** | **1.880-1.914** | **Mo-O-Mo** | **92.41-118.3** |
| **O-O** | **.84** | **Al-O-Mo** | **102.32-104.57** |
| **O-Tm-O (Type-I)** | **64.82-127.59** | **Mo-O-Tm** | **160.21** |
| **Tm-Al27*** | | | |
| **Tm-O** | **2.281-2.409** | **O-Mo-O (Type-i)** | **67.88-107.24** |
| **Mo-O** | **1.685-1.736** | **O-Mo-O (Type-II)** | **148.49-161.17** |
| **Mo-O (Type-II)** | **1.904-1.968** | **O-Al-O** | **84.43-96.08** |
| **Mo-O (Type-III)** | **2.251-2.339** | **Mo-O-Mo** | **91.26-119.59** |
| **Al-O** | **1.882-1.911** | **Al-O-Mo** | **102.5-105.19** |
| **O-Tm-O (Type-I)** | **70.36-124.56** | **Mo-O-Tm** | **160.09-160.22** |
| **O-Tm-O (Type-II)** | **137.12-147.66** |  |  |

**Table S6.** Selected bond distances and angles of all the unreported solids prepared in the study.
