# Supplementary material for: Photoluminescence Properties of Two Closely Related Isostructural Series Based on Anderson-Evans Cluster Coordinated With Lanthanides [Ln(H2O)7{X(OH)6Mo6O18}]•yH2O, X = Al, Cr
Source: Front Chem. 2019 Jan 7;6:631. doi: 10.3389/fchem.2018.00631 (PMC6330572; doi:10.3389/fchem.2018.00631)
Supplement: Supplementary file 2 [file Image.pdf]

Graphical Abstract

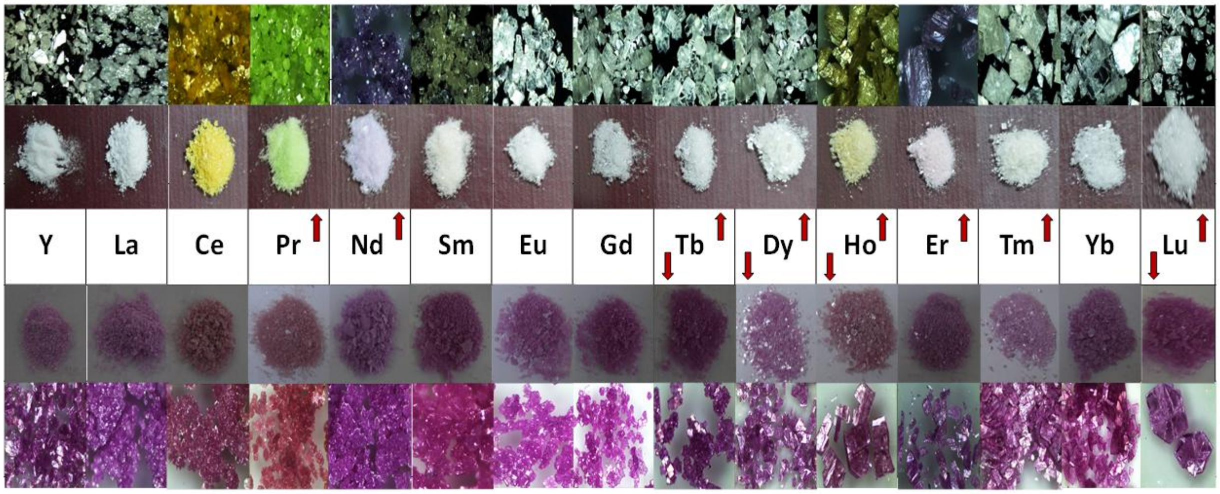

Images of all the solids prepared in the study, taken from a digital camera Nikon D3300 DSLR camera(middle) and Nikon SMZ-745T microscope (top and bottom), the arrows represent solids being reported for the first time.
